# Perceptions and experiences of women referred during obstetric complications in Eastern Uganda: A qualitative study

**DOI:** 10.1371/journal.pgph.0004566

**Published:** 2025-07-14

**Authors:** Richard Keem, Rose Chalo Nabirye, Julius N. Wandabwa, Madeline Powers, Sarah Racheal Akello, David Mukunya, Joshua Epuitai

**Affiliations:** 1 Department of Nursing, Busitema University, Mbale, Uganda; 2 Department of Obstetrics and Gynaecology, Busitema University, Mbale, Uganda; 3 Seed Global Health Uganda, Kampala, Uganda; 4 Mbale Regional Referral Hospital, Mbale Uganda; 5 Department of Public Health and Community Health, Busitema University, Mbale, Uganda; University of Global Health Equity, RWANDA

## Abstract

Women, who are referred, tend to experience several challenges while accessing obstetric care. This study was conducted to explore the perceptions and experiences of women who were referred following emergency obstetric complications. We conducted 17 in-depth interviews with referrals. We explored women’s responses to being referred, what they liked and disliked about being referred, and their challenges during referral. We identified five themes from the data: 1) the facilitators of the referral system, 2) barriers during obstetric referral, 3) the quality of care in the lower-level health facilities, 4) good quality of care in the referral facility, and 5) response to referral and appraisal of the referral system. An early decision to refer, communication between health facilities, and accompanying the woman to the higher-level referral site facilitated the referral process. Financial constraints, poor roads, long distances, multiple referrals, and the use of inappropriate modes of transport were the main barriers to referral. Women experienced disrespect, shortages of medicines and supplies, and unavailability of essential services in the lower-level facilities. The referral site was perceived to provide good quality care for referrals because of warm reception, immediate care, availability of services, and respectful and responsive healthcare providers. Women were fearful and reluctant to be referred because of distant referral sites, failure to recognize obstetric complications, and fear of cesarean section and mistreatment in the referral site. Some women were willing and complied to be referred because of their confidence of care in the referral site, and their ability to perceive obstetric complications. Women experienced challenges with referral systems from poor quality of care, and difficulty reaching the referral site. These experiences and perceptions shaped how women responded to and appraised the referral system. Strengthening the quality of care in lower-level health facilities is critical in improving the referral system.

## Background

Globally, approximately 260,000 maternal deaths were reported in 2023 [1]. The high maternal mortality is partly attributable to delays in decision-making, accessing, and receiving appropriate care [2]. Studies have reported poor maternal and fetal outcomes among women who are referred during labor [3,4]. Obstetric referrals were linked to low Apgar scores, admission to neonatal units, and high risk of perinatal and maternal mortality [3,4]. The poor outcomes among obstetric referrals are attributable to Thaddaeus and Maine’s delays in seeking and receiving healthcare [2,5]. Study findings have shown that a majority (91%) of obstetric referrals experience at least one of the three delays when accessing emergency obstetric care [5].

In Uganda, the healthcare system is divided into four levels of healthcare [6]. The first level of care comprises primary healthcare facilities which provide basic and emergency services [6]. The second level of care includes community hospitals and general or district hospitals that provide basic and comprehensive services such as blood transfusion and cesarean sections [6]. The third and fourth levels are the regional and national referral hospitals respectively that are mandated to provide specialist healthcare services [6]. As a result, the provision of healthcare should begin in the lower levels of healthcare and progress to higher levels of care [6]. Obstetric referrals occur when health facilities without the capacity to manage obstetric complications make referrals to higher facilities [6]. However, referral from the lower level to the next is often not followed as it is common for referrals to be made directly from the first level to the third or fourth level of care [7]. Referrals also tend to occur among health facilities with the same level of care (e.g., from community hospitals to district hospitals) [7]. In addition, patients tend to make self-referrals to higher facilities thereby bypassing primary healthcare services [7]. Ultimately, poor compliance underscores fundamental problems with the referral system in Uganda [8,9].

Women who are referred following obstetric emergency complications tend to experience several challenges when accessing obstetric care [10]. Studies from West Africa have identified communication gaps between the referring facilities, inadequate staffing, shortage of medical supplies and drugs, poor means of transport, and poor road infrastructure as the barriers women experience during referral [10]. In western Uganda, women had unpleasant experiences due to inadequate comprehensive emergency obstetric and care (CEMOC) services in CEMOC facilities, lack of appropriate means of transport to the referral facility, and poor quality of care in the lower-level facilities [9]. Abuse and disrespect of women during referral is common which affects the utilization of maternal and child health services [11,12]. The study was conducted to explore the experiences and perceptions of women who were referred following emergency obstetric complications.

## Methods and materials

### Ethics statement

Ethical approval of the study was obtained from the Busitema University Research and Ethics Committee (BUFHS-2023–86), while Mbale Regional Referral Hospital (MRRH) provided administrative clearance for the study to be conducted in the hospital. We concealed information that could personally identify the study participants. Written informed consent was obtained from the study participants before enrollment into the study. All the participants who were approached agreed to participate in the study. Participants who were below 18 years old were considered as emancipated minors [13]. Written informed consent was obtained from the emancipated minors in line with the guidance from the Uganda National Council of Science and Technology [13].

### Study design and site

This was a qualitative study that used phenomenological study approaches to explore the lived experiences of women following emergency obstetric referral. The study was conducted in Uganda which operates based on a pyramidal referral system (S1 Fig) [6]. Primary healthcare comprises village health teams that are responsible for health education and promotion activities [6]. In addition, primary health centers such as Health Centers II and III provide primary health care services including outpatient services, antenatal care, and conduct uncomplicated skilled birth deliveries [6]. Health centers III are mandated to refer complicated cases to health center IVs or district hospitals [6]. The district hospitals and health center IVs offer CEMOC services such as surgery and blood transfusion in addition to offering basic primary health care services [6]. The district hospitals refer complicated obstetric cases to regional referral hospitals. Regional referral hospitals provide high-level and specialized services, while they may also serve as a teaching hospital and a research center [6]. The regional referral hospitals supervise service provision in the lower-level health facilities [6].

The study was conducted in Mbale Regional Referral Hospital (MRRH). Participants were recruited face-to-face from the postnatal ward, high dependence unit, and neonatal intensive care unit (NICU). The hospital serves as a teaching hospital for the Busitema University Faculty of Health Sciences (BUFHS) and other nursing schools in the region. The hospital receives referrals from the Teso sub-region, Bugisu sub-region, Bukedi sub-region, and Sebei sub-region in Eastern Uganda.

### Study population and sampling strategy

The study population included all mothers who were formally referred from lower-level public and private facilities to MRRH. These included women who were referred during labour because of emergency obstetric complications such as postpartum hemorrhage, prolonged labor, and obstructed labor. We used purposive sampling to select a diverse group of participants who could provide rich in-depth data. As a result, we selected participants from different locations, parity, ethnicity, and birth outcomes. The principle of data saturation guided the selection of study participants. Data saturation was reached after the selection of 17 study participants.

### Data collection procedure and methods

The primary author (RK), a male and a final-year nursing student conducted the in-depth interviews (S1 Text). Although the primary author had limited experience in conducting qualitative interviews, the interviewer was trained on the ethos of qualitative data collection. The senior author supervised the data collection process to ensure the data was rigorous. The interviewer, who was doing the study for purposes of their research thesis, did not have any established prior relationship with the participants. Data collection was conducted between 02^nd^ October 2023–30^th^ November 2023. An interview guide was used for interviewing participants (S2 Text). We collected social demographic data which included age, marital status, level of education, parity, level of referring facility, mode of transport, distance from the referring facility, method of delivery (cesarean section/spontaneous vaginal delivery), and outcome of delivery. Women were asked about their experience following the referral and their reactions to being referred. We further explored what women thought about referral in general, their perception of the quality of care they received, how they were treated, and what they liked or disliked about the referral system. We also assessed the views of women regarding the challenges that they faced in reaching the referral facility. The interviews were audio-recorded and they lasted 16 minutes on average. Interviews were conducted in a separate and private room in the hospital to ensure confidentiality and privacy. Field notes were not collected during the interview process.

### Data analysis

Transcription was done verbatim. The first and last authors conducted data analysis manually without the use of software. Inductive thematic analysis, as described by Braun and Clarke, was used for data analysis [14]. Thematic analysis was appropriate for the study as its flexibility aligned with the epistemological underpinnings of the study [14]. Furthermore, thematic analysis was chosen in the study because of its ability to summarize key features of the study [14]. In the first step of thematic analysis, transcripts were read several times to understand and generate initial ideas about the data [14]. Subsequently, we identified initial codes from the dataset [14]. The codes represented the basic elements of the dataset which captured the essence of the study [14]. Codes were identified manually and inductively from the data [14]. The next phase involved the identification of themes which comprised grouping and collating the initial codes [14]. The identified themes were reviewed, refined, and renamed to capture the recurring patterns in the data [14]. The last phase of thematic analysis involved the writing of a report to provide a comprehensive understanding of the perceptions and experiences of women during referral [14].

## Results

### Description of the study participants

The majority of the participants were from more than a 5km radius of the referral facility and had contacted the referral facility to inform them of the incoming referral, while only 29% of the women had used an ambulance to come to the referral site (S1 Table).

### Theme 1: Barriers during the obstetric referral process

Participants identified challenges of reaching the referral hospital. The challenges included financial constraints, long distances, poor roads, modes of transport, and physical discomfort (S2 Fig).

#### Subtheme: Financial constraints.

Financial constraint was the major barrier that women faced during the referral process. Money was required to cater for the high costs of transport, while money was needed to buy food, medicines, and welfare while in the referral facility. The emergency nature of the referral gave women no time to look for money. As such, some had to wait for the male partner to bring the money. Some of the participants had to borrow money from their significant others which process delayed the time to reach the referral facility.

*“We did not have enough money for transport, food, and for staying here. My mother had to make a call to some people to borrow us, we had to borrow some and finally we got the money.” (IDI 3, 27-year-old Parity 3+0).*

#### Subtheme: Long distance and poor road infrastructure.

The long distance and poor road infrastructure were challenging for some women. The majority of the women were referred from distant facilities where 47% (8/17) of them were 20km away from the referral hospital. Pregnant women traveled for long distances to reach the referral facility. The weather conditions and hilly terrain conditions on the road worsened the already poor road infrastructure. This delayed women from reaching the referral hospital, while it also made the journey to the referral facility to be more painful.

*“I was feeling very bad on the road because I used a motorcycle. First it had rained and the road was very bad…the humps made the pain even more along the road” (IDI 10, 23-year-old, parity 1).*

#### Sub-theme: Inappropriate means of transport.

Few women used ambulances compared to a majority that used motorcycles and other public means of transport to reach the referral facility. The ambulances were perceived as costly, while they were sometimes unavailable, or dysfunctional to transport women to the referral facilities.

*“We came with a boda-boda [motorcycle] here but I was worried on the way because the humps might make the baby come very fast…..also the water [draining liquor]was coming so we took long on the road.” (IDI 9, 16-year-old parity 1)*

#### Sub-theme: Multiple referrals.

Women faced challenges of multiple referrals from one health facility to another. Multiple referrals occurred when women were referred to CEMOC facilities which happened to lack the capacity to provide the services, especially cesarean section. This resulted in a series of back-and-forth referrals.

*“I did not like referrals because of the many referrals from here to there. They could not make one decision of one hospital where I can get the service at once.” (IDI 11, 20-year-old parity 1)*

#### Sub-theme: Poor hygiene in the facilities.

Poor hygiene in the referral facility was another challenge that women faced during the referral.

*“What I did not like? The referral was good but here! There is some smell outside there where we sit. The cleaning is not okay outside the ward. People urinate out at night. There is also some blood near the rubbish bucket.”* (IDI 9, 16-year-old parity 1).

### Theme 2: Poor quality of care in the lower-level facilities

#### Subtheme: Shortage of staff and medicines.

The care in the lower-level referring facilities was seen to be substandard due to shortages of staffing, and the unavailability of medicines and or medical supplies. It was often common to find one midwife in a health center III who was serving a disproportionate number of women, while doctors/anesthetic personnel may sometimes be unavailable in CEMOC health facilities, especially during weekends. Besides a shortage of personnel, there was also a shortage of basic and essential drugs and medical supplies (e.g., gloves), especially in the peripheral facilities. Ultimately, the shortage of staff and medicines delayed the onset of treatment but it also affected the provision of CEMOC services, especially emergency cesarean section. Although mothers were referred because of obstetrical complications, the shortage of staff and medicines were often the underlying primary reasons for referral from CEMOC facilities that could provide CEMOC services. This led to the inundation of the high-level referral facilities with unnecessary referrals but also made the healthcare workers in the regional referral hospital reluctant to receive referrals from CEMOC facilities.

*“They told me the doctor is not there more over it is called health center four! Doctor is supposed to be there.” (IDI 8, 35-year-old Parity 5+0)*

*“When we reached…, the doctor was there but the challenge we got was they never had medicine in the theatre so they told me we have to refer you again.” (IDI 4, 29-year-old Parity 3 +0).*

#### Sub-theme: Lack of essential services.

The shortage of staff and medical supplies resulted in a lack of provision of basic and essential services. This necessitated referral of the woman but also resulted in referral without provision of any basic treatment. One woman overheard how the healthcare workers at the site she was referred to were surprised by the poor quality of care in the facilities that were making the referrals.

*They were complaining…[saying] how can you send someone when she is badly off like this? …how can a patient come and you don’t work on them? Then, you send for us…those people are not doing good (IDI 8, 35-year-old parity 5).*

#### Sub-theme: Disrespect of women.

Care in the lower facilities was perceived to be of poor quality, and tainted with disrespect for women. This was in the form of verbal insults, discrimination, and abandonment during treatment. The disrespect stemmed from low staffing and shortages of medicines and medical supplies. For example, the shortage of medicines meant that women had to buy them, and as such, care was given to only women who could buy the required medicines. Those who could not afford were abandoned without receiving treatment.

*“When you have money, they work on you very fast but when you don’t have money, they leave you there…immediately you reach there like this, they say give the money for gloves. When you don’t have, they don’t work on you…”* (IDI 8, 35-year-old, parity 5).

*“When the time reaches for treatment…..they will start telling you bad words. The response is like aah, we are tired. You people are also disturbing us. … And sometimes they can just jeer at you... So that thing hurts me a lot” (IDI 11, 20-year-old, parity 1)*

Disrespect also occurred in the form of threatening to refer women who were assertive or those who were seen as non-compliant. Healthcare workers used threats that mistreatment would continue or even become worse for women who were referred to the regional referral hospital. This made women to have low expectations of the referral hospital to which they were referred.

*“I did not like the way some nurses can scare mothers putting a bad picture ahead of the place they are referring you to. Maybe to discourage you…‘like, this one is bragging, we have to refer her’.” (IDI 11, 20-year-old, parity 1)*

*“I came with that mind of these people don’t care but I was surprised they care was not what they told me.”* (IDI 8, 35-year-old, parity 5).

Although women experienced disrespect at the lower facilities, some were treated with respect. Some healthcare workers treated women well with dignity, encouraged asking questions, and were friendly to the women.

*“The care of the mothers there [lower-level facilities] is not bad. Even the healthcare workers are not bad because they do not talk badly. They are friendly with mothers as they care. They ask you and they treat you very well.” (IDI 11, 20-year-old parity 1).*

### Theme 3: Good quality of care in the referral facility

#### Sub-theme: Warm reception and immediate care.

Almost all (15 out of 17) women thought that the quality of care in the facility they were referred to was of good quality. This was because women were warmly welcomed and cared for immediately upon arrival at the health facility. However, some of the women thought care in the referral facility was not good related to delays in receiving care. Nevertheless, the majority appreciated the continuous monitoring to assess their well-being and that of the baby. Women appreciated the triaging system in the hospital which enabled those who were badly off to be treated first, while those who were scheduled to undergo Caesarean section were operated on time. Women, who were reluctant to be operated on and wanted to deliver normally, appreciated that they were given enough time to push. This validated the healthcare provider’s decision to operate on them.

*“Here the care is good because within two hours they were able to take me to the theatre to operate since the baby was not doing well. Even the nurses and doctors keep checking on you every time to make sure that you are attended to…”(IDI 1, 23 year old, parity 1).*

*“For sure, it was not good because they took long to attend to me….But the care later was good because the operation went well and they keep monitoring you while on the ward, when you report any pain, they attend to you immediately.” (IDI 15, 30-year-old, parity 2).*

#### Sub-theme: Availability of healthcare providers, medicines, and CEMOC services.

Unlike at the lower facilities where there was a marked shortage of staff and essential medicines, women thought that the care in the facility they were referred to was good because of the availability of healthcare providers and essential medicines. Healthcare providers were supplemented by the presence of medical or nursing students who were always available to attend to the needs of women. Although some medicines and supplies in the referral facility were not available, this was not seen as burdensome to women. The CEMOC services such as functional theatre services were also available.

*“From here, the doctors and nurses are both doing well because they give you treatment in time and medicines are available except some special ones, they tell you to buy.”* (IDI 2, 20-year-old parity 2).

*“The care is so nice because when you call…the healthcare provider is ready for you all the time. There are also these students, they even help us when the healthcare workers are busy…they are always there. You have someone to call and they respond fast.”* (IDI 4, 29-year-old, parity 3).

#### Sub-theme: Responsive and respectful healthcare providers.

Besides the availability of healthcare providers, women viewed that the care was good because the healthcare providers were responsive to their needs, were concerned and respectful, and encouraged them to ask questions. The respectful care occurred in the form of consented care, information sharing, non-discrimination, and respectful interaction. The healthcare providers reassured, comforted, and encouraged women during labor. This was surprising to women because they anticipated, from rumors, to be mistreated in the facility they were referred to.

*“The care here is good because nurses and doctors are concerned. They kept checking and asking how you are feeling and they respond when you call for help.”* (IDI 5, 27-year-old, parity 5).

*“The care here is good because they are caring for me and I also see them caring for other mothers equally. The nurses and doctors talk very well.”* (IDI 7, 32-year-old, parity 3).

### Theme 4: Facilitators of obstetric referrals

#### Sub-theme: Communication between referral facilities.

Women reported that some healthcare providers called their counterparts in the receiving facility to inform them of an incoming referral. Women thought that informing the healthcare providers in the receiving facilities enabled them to receive immediate care upon arrival, while it also facilitated the smooth handover of the referral patients.

*“My experience has been good because the doctors advised me well about why I have to go to a referral hospital. And also, they made calls to make sure I was going to be attended here when I arrived so they care for me well.” (IDI 3, 27-year-old, parity 3).*

#### Sub-theme: Healthcare providers escorting women to the referral facility.

Some of the healthcare providers escorted women to the referral facilities. Accompanying the women to the referral facilities enabled them to receive reassurance and comfort from healthcare providers during transit, but it also enabled them to receive care immediately upon arrival.

*“The way the nurses comforted me during the way to make sure that my pain was minimal and they kept on encouraging me to take heart…” (IDI 4, 29-year-old, parity 3)*

#### Sub-theme: Timely decision-making to refer to early.

Timely and early referral of women with complications enabled women to receive appropriate care at the referral sites. Women considered the healthcare providers to be caring because they decided to refer them early.

*“I think they are caring since they also decided to say let us take this one for operation and they even accompanied me to make sure they attended to me very fast. And when we reached here, they really cared and looked for the doctor”* (IDI 1, 23-year-old, parity 1).

### Theme: Response to being referred and women’s appraisal of the referral system

Considering the challenges that women anticipated following referral and their experience of poor quality of care in the lower-level facilities, women responded differently after being referred. Some were fearful, anxious, and reluctant, while some were willing and confident in the quality of care they were to receive in the high-level referral facilities. At the end of the referral process, women positively appraised the referral system.

#### Sub-theme: Fearful and reluctance to be referred.

Women expressed anxiety, fear, and uncertainty when they were informed that they were to be referred. Emotional distress was heightened by concerns about the baby’s well-being, fear of poor birth outcomes, and fear of cesarean section. Women thought that referral meant that they were to be operated on. This made them to be more anxious, especially among those who had a previous scar and were hoping to achieve a normal delivery. Some of the women were anxious about the alleged disrespect in the referral health facilities though this was not the case.

“*At first, I feared. So, I was scared. I hear people there complaining about the nurses of MRRH that they are difficult [abusive and disrespectful]. But me I have seen them; they are not what I hear of and expected of them.” (*(IDI 12, 29-year-old, Parity 4).

Some of the women were reluctant to comply with medical advice to be referred which stemmed from the mother’s failure to recognize the obstetric complication and the need for referral, fears of mistreatment in the receiving facility, fears of Caesarean operation, and the woman’s preference for spontaneous vaginal birth. The severe labor pains forced some women to comply with the advice to be referred. Although women were willing to be referred, healthcare providers in some facilities were reluctant to refer women. Some of the women had to request to be referred.

*“It was automatic, I thought of an operation. I refused to come on the very day they referred me; thinking things would be fine but instead, there was no change. I insisted….I have to push my baby. I had to go back home. Two days after, the pain was severe so I had to come back straight here.” (IDI 6, 22-year-old, parity 1)*

#### Sub-theme: Willingness for referral.

The majority of the women expressed willingness to be referred. This was because of the extreme pain, the poor progress of labor, ominous signs (e.g., meconium staining, decreased fetal movements), lack of alternatives, and the likely poor birth outcomes which motivated and validated the need to comply with the advice. Confidence in the hospital where they were referred further cemented their willingness to come to the referral facility. This was because the referral facility was perceived to have the capacity to manage their condition.

*“I was willing to come because now the baby was not coming but I had labored for long…I began to worry that I may lose my life and the baby as well”* (IDI 3, 27-year-old, Parity 3).

*“When I was told about Mbale Regional Hospital, I had the confidence because I knew it was a big hospital…I knew things would be alright for me and baby so I had to accept because there was no option now….” (IDI 4, 29 years old, Parity 3).*

#### Sub-theme: Positive appraisal of the referral system.

When women were asked what they liked about the referral system, a majority positively appraised it because of the resulting positive birth outcomes. Women, who were initially worried about losing their lives and that of the baby, were overjoyed with the positive birth outcomes following referral. The positive outcomes made women understand the need for referral.

*“I have liked it because now I am going back with someone that I was not expecting…I knew it was dead. If I was not to come, I would have probably lost my baby and myself*.” (IDI 1, 23-year-old parity 1)

***“*** *My overall experience has been good because finally, I came to understand that the healthcare worker was right to refer me in the first place, but, I thought I was able to manage to push not until things did not work the way I wanted.” (IDI 6, 22-year-old, parity 1)*

However, some participants did not like being referred because of the costs of transport, long distances, and out-of-pocket expenditures.

*“I have not liked referral. Again, because of transport money. I come from very far. I put in transport…to and from. So, I have not liked that because at…[Name withheld], I just have to go to the health center at no cost.” (IDI 15, 30-year-old, parity 2).*

## Discussion

The study was conducted to explore the experiences and perceptions of women who were referred during delivery. Women noted barriers and facilitators of the referral process. Care in the lower-level facilities was rated poorly, while care in the referral facility was rated positively. Women reacted differently to being referred based on their perceptions of the birth outcomes, experiences, and expectations of care in the receiving facilities.

Our study identified barriers to the referral process, especially financial constraints and transportation costs to the higher referral facility. Referral made women travel to distant facilities which heightened the need for money to cater for costs of transport, welfare, and out-of-pocket expenditures while in the referral facilities. Women resorted to borrowing, which was similar to study findings from a West African country where women had to wait for the male partner, who was always away from the facility, to bring the money [15]. The high costs of transport, poor road infrastructure amidst poor weather conditions, and long distances further underscored barriers to accessing referral facilities. Like in other studies [5,16], financial and transportation challenges made women resist referral. These barriers could contribute to delays in reaching referral facilities and poor obstetric outcomes among referrals. Most of the participants used motorcycles and public vehicles because the ambulances were unreliable, unavailable, expensive, or dysfunctional, a finding that was reported in other settings in low-and middle-income countries [9,15,17]. Use of inappropriate means of transport negatively affects the woman’s privacy in public vehicles, continuity of care during transit, and it ultimately delays the time in reaching the referral facility [17].

Consistent with the poor quality of care in the lower-level facilities reported in the previous studies, our study findings equally underscored the poor quality of obstetric care in these health facilities [9,11,17]. The poor quality of care was attributable to shortages of staffing, medicines, and supplies. Although emergency obstetric complication was the primary reason for referral, in some cases non-obstetric reasons (e.g., absence of a doctor to operate) could have been the underlying reasons for referral in facilities that could provide emergency cesarean section services [9]. Similar studies have noted the lack of a doctor, anesthetist, power outages, and medicines in the theatre as the primary reasons for referral [9,11,17]. The lack of healthcare providers was especially reported during weekends which suggests healthcare providers may have traveled to be with their families in the urban settings. In our study, women were concerned by the multiple referrals due to the deficiencies in the provision of CEMOC services in the lower-level facilities. Poor quality of care in the lower health facilities decreases the community’s confidence in the referral system leading to bypassing of the peripheral facilities and consequent overcrowding in higher-level facilities [2,8]. Addressing the challenges of low staffing and shortages of medicines and supplies can improve the quality of care in the lower referring facilities.

Similar to the poor quality of care noted in the lower health facilities, women who were due for referral experienced disrespect in the lower-level facilities. The forms of disrespect extended to the use of threats to refer women who were *“non-compliant”*, but it also led to rumors that disrespect would continue or even become worse in higher-level facilities though this was not the case. Our findings resonate with findings from western Uganda, where women experienced mistreatment during the referral process [9]. In our study, disrespect was perceived to emanate from a shortage of personnel where the healthcare providers were overwhelmed, while the requirement to buy medicines resulted in discrimination and abandonment. Evidence has underlined the negative influence of disrespect in reducing utilization of skilled birth attendance, negative birth experience, and risk of mental health problems [12,18,19].

While women thought that the care in the lower facilities was poor, they reported experiencing good quality of care in the referral facility. This was related to the availability of healthcare providers supplemented by the presence of nursing/medical students, the availability of CEMOC services including a functional theatre, and respectful and responsive providers. Women were surprised to receive respectful maternity care in the referral facility contrary to rumors of prevalent mistreatment in these settings. The availability of functional CEMOC services and respectful maternity care boosts confidence for women to come to the regional referral facility and results in positive birth outcomes [15,20].

In our study, some of the women reported that healthcare providers informed their counterparts in the referral facility of the incoming referral, while some even escorted them to the referral facility. This was contrary to findings in the literature which underscored critical challenges in lack of communication, feedback, and escorting women to the referring facilities during the referral process [10,11,17]. In western Uganda, midwives were unwilling to escort women to the referral facility because the public vehicles used would not bring them back to the facility [11]. Women who were escorted during the referral process could have been from a private health facility because these facilities prefer to accompany women during referral [21]. Informing higher facilities of the incoming referral and escorting them to the facility was seen to facilitate handover, and result in immediate provision of healthcare on arrival.

Our study noted a varied response of women to being referred which ranged from reluctance and fear to willingness to be referred. Challenges with referral, poor quality of care, anticipation of good quality of care in the receiving facility, and being escorted to the referral facility shaped how women responded to being referred. In Ghana [22], women were reluctant to be referred mostly because of financial implications, a finding that was consistent with our study. Our findings were consistent with studies that cited fear of operation, distant referral facilities, mistreatment in the referral sites, and failure to recognize obstetric complications as the major reasons for reluctance to comply with [10,22]. Women complied with the advice to come to the referral hospital because of the extreme pain, ominous signs, and fear of poor birth outcomes. The confidence in the quality of care they would receive in the regional referral hospital further reinforced their commitment to come to the regional referral facility. Confidence in the referral facilities highlights the critical role of quality care in encouraging skilled birth attendance. Ultimately, positive birth outcomes following a referral made women positively appraise the overall referral system but it also validated the decision for referral. This underscores the impact of timely referrals in promoting positive birth outcomes.

## Study limitations

The study was conducted in a regional referral hospital. The experiences of women in our study may not be transferrable to other settings with different populations and characteristics. The study was conducted in a hospital setting which could have resulted in social desirability bias and reluctance to disclose negative experiences of care. The study findings are limited to only women with formal referral letters. Consequently, our findings do not capture the experiences of women who were self-referrals or those who did not have formal referral letters. In addition, the findings do not capture the experiences and perceptions of healthcare providers regarding the referral process in Uganda. Future studies could explore experiences from diverse perspectives regarding the referral process during delivery.

## Conclusions

Women experienced challenges during referral related to barriers to accessing the referral facility such as financial constraints, long distances, poor roads, lack of appropriate means of transport, multiple referrals, and poor hygiene. Quality of care in the lower-level health facilities was seen to be poor related to disrespect of women, inadequate medicines, supplies, and CEMOC services. Women perceived care in the higher-level facility to be of better quality because of the warm reception on arrival, provision of immediate care, availability of basic and CEMOC services, and responsive and respectful healthcare providers. Early referrals, improved communication between the referral facilities, and healthcare providers escorting women to referral sites facilitated the referral process. The challenges of reaching the referral facility, quality of care, and good practices of referral shaped how women responded and appraised the referral system.

## Supporting information

S1 Fig Referral system in Uganda (adapted from the Ministry of Health Uganda [6]). (TIF)

S2 Fig Experiences and perceptions of women referred following obstetric complications. (TIF)

S1 Table Description of the study participants. (TIF)

S1 Text^‌‌^ (DOCX)

S2 Text (DOCX)
