# Supplementary figures and images for: Perceptions and experiences of women referred during obstetric complications in Eastern Uganda: A qualitative study
Source: PLOS Glob Public Health. 2025 Jul 14;5(7):e0004566. doi: 10.1371/journal.pgph.0004566 (PMC12258546; doi:10.1371/journal.pgph.0004566)

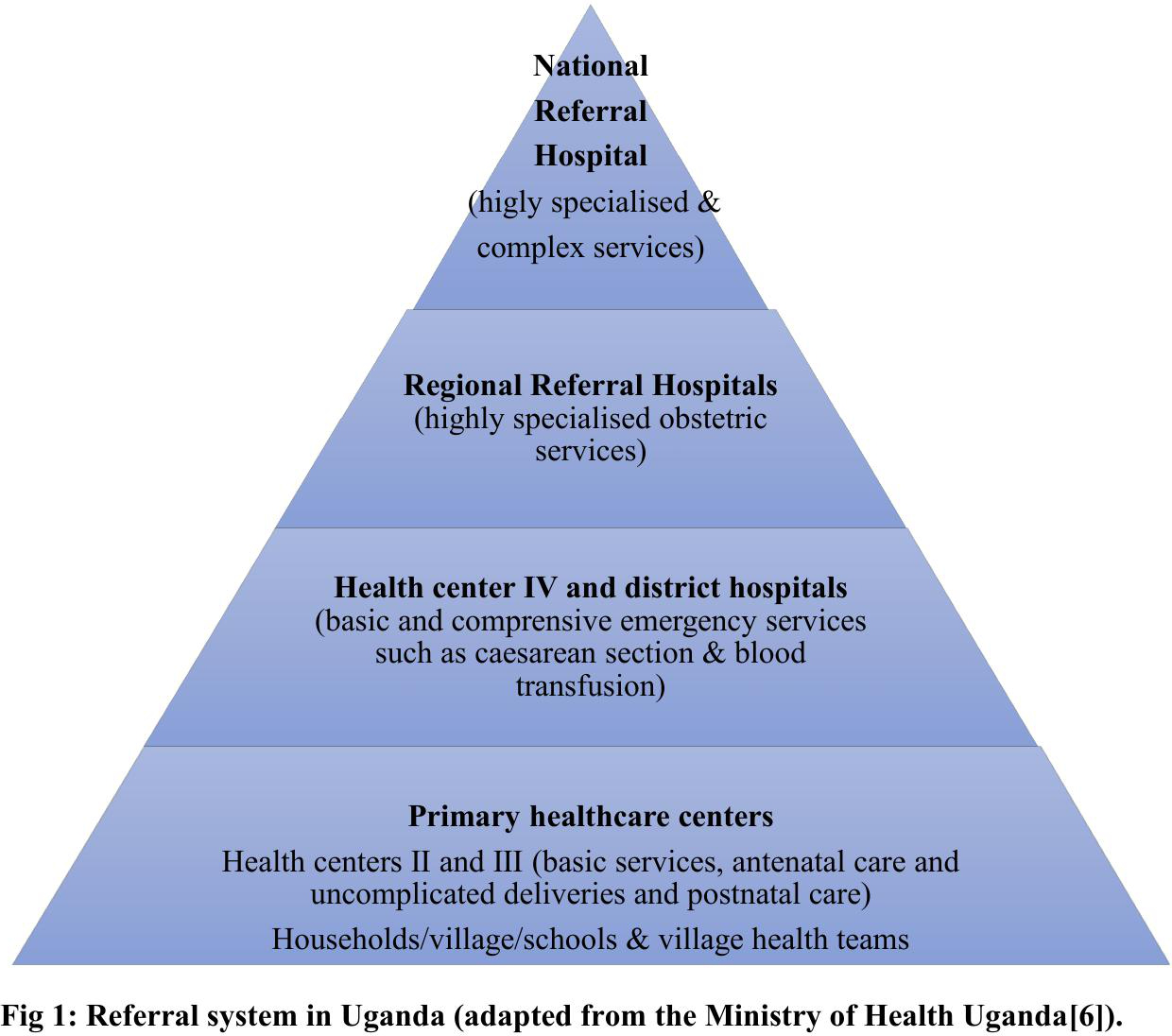

Supplement: S1 Fig — (TIF) [file pgph.0004566.s001.tif]

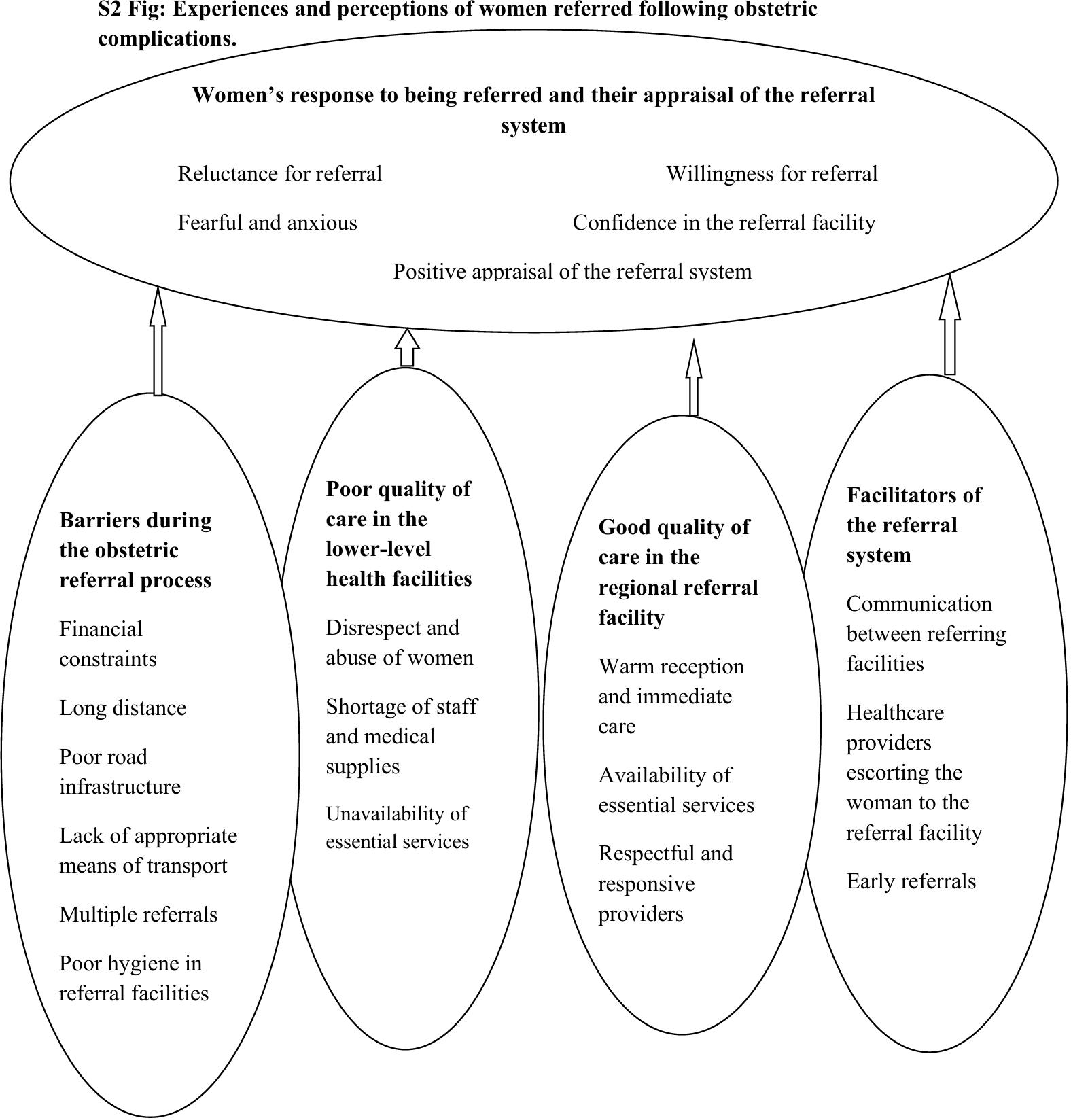

Supplement: S2 Fig — (TIF) [file pgph.0004566.s002.tif]

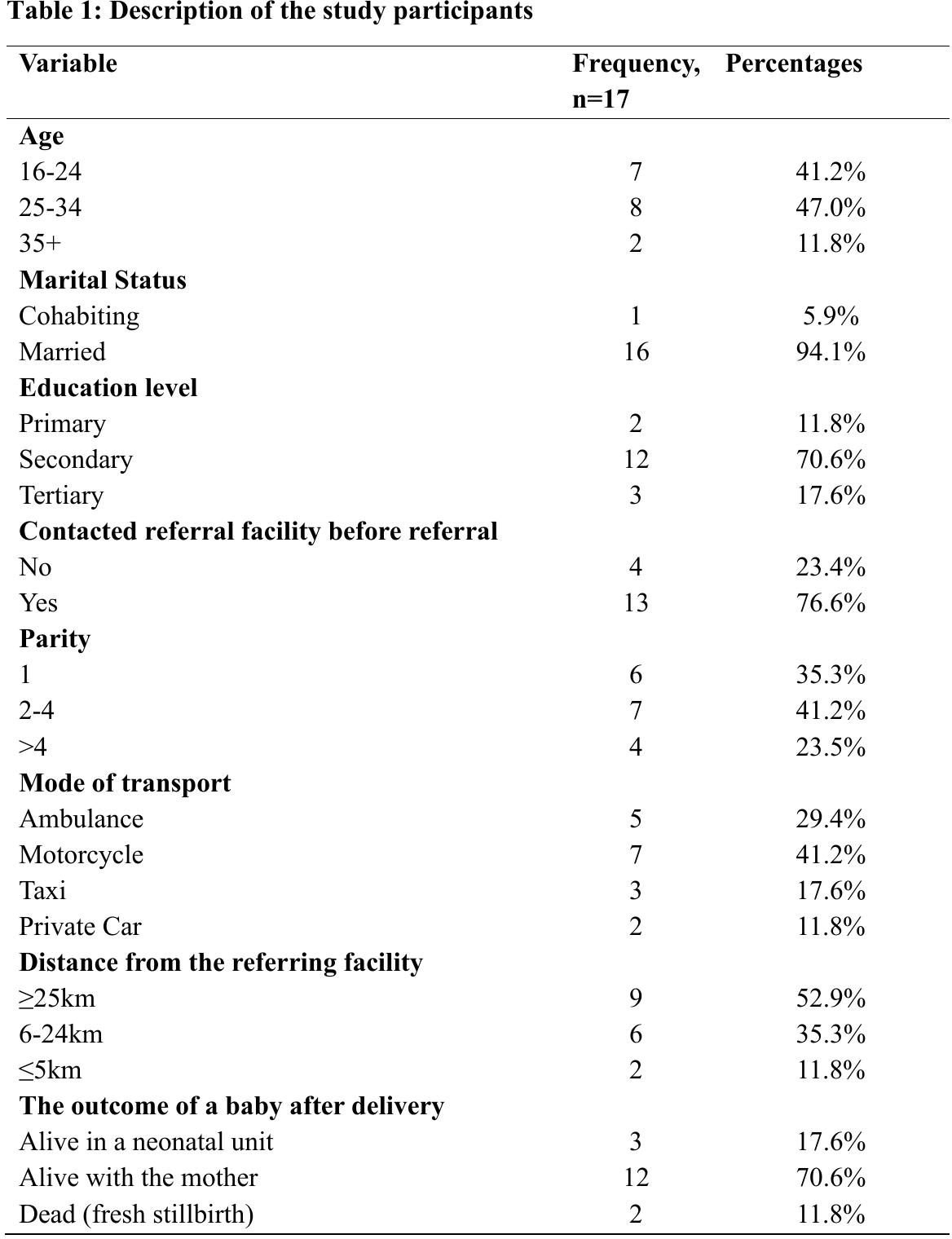

Supplement: S1 Table — (TIF) [file pgph.0004566.s003.tif]

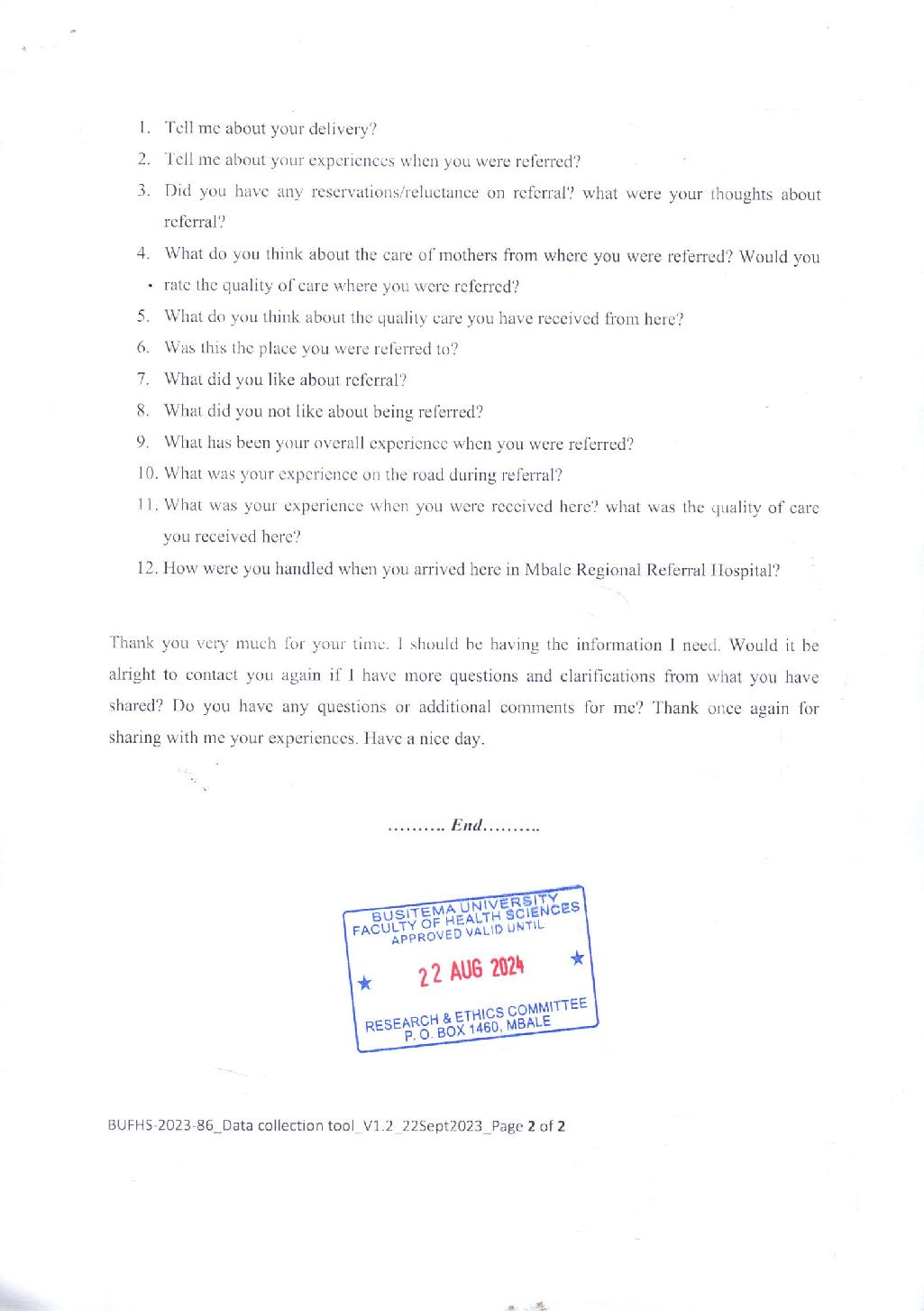

Supplement: S2 Text — (DOCX) [file pgph.0004566.s005.docx]
