# Supplementary material for: Perceptions and experiences of women referred during obstetric complications in Eastern Uganda: A qualitative study
Source: PLOS Glob Public Health. 2025 Jul 14;5(7):e0004566. doi: 10.1371/journal.pgph.0004566 (PMC12258546; doi:10.1371/journal.pgph.0004566)
Supplement: S1 Text‌‌ — (DOCX) [file pgph.0004566.s004.docx]

**INDEPTH INTERVIEW Transcripts**

**INTERVIEW DETAILS:**

**Interview time:** 22 minutes:18 seconds

**Interview location:** Postnatal ward (.......)

**Moderation language:** English

**Participant number:** 01

**Conversation language definitions:**

**Bold:** Title/interview questions

**Normal text:** Respondent

**Silent talk:** (Pause)

**I:** Interviewer

**R:** Respondent/participant

**Q:** Interview question

| Demographic data: | Response |
| --- | --- |
| Initials  Age:  Marital status: Married  Education level  Parity  Source of referral  Contacted the receiving facility prior to referral  Mode of transport  Distance from the referring facility  Mode of delivery  Outcome of baby after delivery | NV  23 years  Married  S.4  1  HC-IV  Yes  Ambulance  28 Km  Caesarean section  Alive with the mother |

**RECORDED INTERVIEW**

**Interviewer: Q1 Tell me about your delivery?**

**Respondent 1:** “My delivery? I started labor pains on Friday, that night I was not feeling well, I was like aah it maybe it is stomach pain oba what? So, I decided to keep quiet for some time. I told my mother when it was worse when I started seeing some blood and some water coming out so that is when I told her then we went to the hospital, they checked me and they said ‘mbu it is time but what has not yet opened?’ I waited and waited, then they checked me again, they said ‘they were like aah now it is 4 cm’ and I kept on waiting. I waited the next day, the labor pains disappeared on Sunday, the pains disappeared. I told mammy now the pains have disappeared, maybe it was just stomach pain why can’t we tell them to discharge us, I will come back when it comes back again because I slept nothing, the following day we were just there and they agreed that okay you go since you are near. So, when they told us, mammy was like let us first take tea before we go home. So, we were seated outside. Then I felt back pain while taking tea but my mum didn’t see me but those nurses saw me and they were like no you want to go home when this child is feeling pain. They checked me and were like am in 6 cm so don’t go home but am not feeling any pain, I said okay! We slept there again, now at night I felt back pain that all night back pain, back pain and increasing. In the morning again stomach pain and the nurses were like maybe it was infection and they told me to take some medicine and go home, but I said how can I go home when am not feeling well, I was like you checked me and you told me I was 6 cm, now why should I go home? I told my mum since now our nurses are there, we can go, we went and I was feeling pain. My mother said you come and we go and they check you. They checked me and they are like an I 7 cm mbu you go back the child is very big, I cannot keep you here since the child is the first one, we don’t have any doctor to operate you, you have to be near where they can operate you. The nurse was good and said no let me call them and tell them this person is not fine. So, the nurses said let her come, they checked me that night and it was now 8 cm. I was not feeling well, the stomach disturbed me, that Tuesday the stomach disturbed me a lot, the nurse was like you move ko-around for the gundi to first bring water. They were like the head has shown but the water is not yet. I reached time and I told mummy am going to die if you keep n waiting for that, I said you go and talk to them, she went and talked to them and they said you come and we check you. Reaching there, water came which was very dirty, green. They were like, this child is tired, is not breathing well. For sure the baby was not even moving in the stomach. They said we have to get an ambulance very quickly to take you because the doctor is not there to operate you, he was there in the morning and left early so that is how I came to ....... we came with the nurse of there, they escorted me and they worked on me quickly”.

**Interviewer: Q2 Tell me about your experiences when you were referred?**

**Respondent 1:** “Anti- when they told me that am going to be referred, they asked me is the baby moving, I said no. so for me I came knowing the baby is dead, because I came with that pain, it was really painful, pain! I was feeling like something was pushing down to come out, so me I knew now the baby is dead already.”

**Interviewer: Q3 When you were told, you are coming to ......., did you have any reservations/reluctance on referral? What were your thoughts about referral?**

**Respondent 1:** “Anti- we did not have enough money for transport, food and for staying here. My mother had to make a call to some people to borrow us, we had to borrow-ko and finally we got the money.

I didn’t have any reservations; I was willing to come because the pain was a lot and I was thinking now am dead the pain (pause) hmmm! And I did not know that I will even reach ........ This child was very tired because when they removed from the stomach, the cry was not okay, just crying slowly. People out did not hear the cry and that worried me again.”

**Interviewer: Q4 What do you think about the care of mothers from where you were referred? Would you rate the quality of care where you were referred?**

**Respondent 1:** “I think they are caring since they also decided to say let us take this one for operation and they even accompanied me to make sure they attend to me very fast, and when we reached here, they really cared and had to look for the doctor and they left knowing that they had done a big part and they left (pause) in terms of medicines, other medicines they tell you to buy mbu it is not there. Others they give it to you. I can say the care was fair because they took time to decide to make a referral not until the dirty water came.”

**Interviewer: Q5 What do you think about the quality of care you have received from here?**

**Respondent 1:** “the care here is good also ooh. It is good because I see they can come and ask how you are feeling now. They are like ‘are you seeing any improvement?’ Like that, they come to treat you, they want to be aware about the mother’s condition. Also medicine we are buying some and some they give from here.”

**Interviewer: Q6 Was this the place you were referred to?**

**Respondent 1:** “Yes this was the place I was referred because it is near my district and the nurses even called here before we left HC-IV.”

**Interviewer: Q7 What did you like about referral?**

**Respondent 1:** “About referral? I have like it because now I am going back with someone that I was not expecting because by the time the water was dirty and the baby was not moving, I knew it was dead. If I was not to come, I would have probably lost my baby and myself. I have also liked the care given to mothers who are transferred they really make sure they inform the doctors before you leave.”

**Interviewer: Q8 What did you not like about being referred?**

**Respondent 1:** “hmm, have not moved so much that have learnt somethings around, that one I don’t know because since operation, I have been just inside and also it is my first time to come here to ....... Hospital. The other thing I did not liked about referral is, it is far from home and we had to borrow money because they say that if you want to save life, you have to put in money. Now if we didn’t get a debt there, means I could have died. Now you go and pay knowing that it did work and helped.”

**Interviewer: Q9 What has been your overall experience when you were referred?**

**Respondent 1:** “The transport is also high because ambulance we paid 70,000/= from ....... to here so that they rush quickly to buy fuel so that they bring you quickly. But the part of the care was good.”

**Interviewer: Q10 What was your experience on the road during referral?**

**Respondent 1:** “From ......., the road is bad until some distance then you join the tarmac, but at least the driver was good and the nurses were comforting me in the ambulance till we reached here. But I was worried If I was going to reach here because the pain was too much and the baby was not moving.”

**Interviewer: Q11 What was your experience when you were received here? What was the quality of care you received here?**

**Respondent 1:** “here the care is good because within 2 hours they were able to take me to the theatre to operate since the baby was not doing well, even the nurses and doctors keep checking on you every time to make sure that you are attended to, since it is my first time, I don’t know much but that is what I have seen.”

**Interviewer: Q12 how were you handled when you arrived here in ....... Regional Referral Hospital?**

**Respondent 1:** “They handled me well because I was feeling a lot of pain and even making noise but they were not hard on me. They had to hurry working on me, they also told the parents to buy other things for the theatre. they worked on me within two hours they took me to the theatre, and here I have my baby.”

**INTERVIEW DETAILS:**

**Interview time:** 16 minutes:14 seconds

**Interview location:** Postnatal ward (.......)

**Moderation language:** English

**Participant number:** 02

**Conversation language definitions:**

**Bold:** Title/interview questions

**Normal text:** Respondent

**Silent talk:** (Pause)

**I:** Interviewer

**R:** Respondent/participant

**Q:** Interview question

| Demographic data: | Response |
| --- | --- |
| Initials  Age:  Marital status: Married  Education level  Parity  Source of referral  Contacted the receiving facility prior to referral  Mode of transport  Distance from the referring facility  Mode of delivery  Outcome of baby after delivery | SK  20 years  Married  S.4  2  HC-IV  Yes  Ambulance  28 km  Caesarean section  Alive with the mother |

**RECORDED INTERVIEW**

**Interviewer: Q1 Tell me about your delivery?**

**Respondent 2:** “my delivery was not easy! it was around 4pm when I went to HC-IV, they examined me and said I have to be transferred to RRH because the doctor who works in the theatre is not there. So, they referred me to RRH. I reached here at around 12 am, they received me and examined me and they said my Uterus is at 6 cm (Cervix) that was around 1 am. They repeated the examination at around 9 am and they said my uterus was at 5 cm then again, they repeated the examination at 12pm and they told me it was still at 5 cm. the final doctor came and examined me at around 3 pm and they told me that the baby is big, I have to go to theatre for operation. That was my information about my delivery.”

**Interviewer: Q2 Tell me about your experiences when you were referred?**

**Respondent 2:** “I was experiencing a lot of pain and I was to go to the theatre and also thinking that I will go to the theatre because they said that ‘the doctor who works in the theatre is not present’ and I felt bad and they told me ‘You have hope to give birth but it is half-half so I have to go to RRH for operation’.”

**Interviewer: Q3 When you were told, you are coming to RRH, did you have any reservations/reluctance on referral? What were your thoughts about referral?**

**Respondent 2:** “I had some feelings, a lot of feelings. I felt so bad because I was expecting may be this time, I can try to give birth, but it resulted into operation again. I just left everything to God to take control. I also said now am going to have 2 scars, means this was the only chance to try, no more trying to push again. The good thing is that, the ambulance was free and they did not tell us to pay any money for fuel

**Interviewer: Q4 What do you think about the care of mothers from where you were referred? Would you rate the quality of care where you were referred?**

**Respondent 2:** “Hmm, the care? anyway I don’t know much since it was my first time to come to health centre IV. From the time I was there, they were doing so well, because (Pause) when I reached, they took care of me like a patient, they listen to you, they talk to you and they advise you, they make you to have hope, not to worry in your life. Therefore, to me the care was excellent.”

**Interviewer: Q5 What do you think about the quality of care you have received from here?**

**Respondent 2:** “From here, the doctors and the nurses are both doing well, because they give you treatment in time and the medicines are available except some special once they tell you to buy, they also give you time to try to push, if you fail then you go to the operation. When they see that you are strong enough either you are having any previous scar, they first give you time like me I had enough time but the child refused t come.”

**Interviewer: Q6 Was this the place you were referred to?**

**Respondent 2:** “yes this was the place I was referred; they did not tell me of any other place.”

**Interviewer: Q7 What did you like about referral?**

**Respondent 2:** “I liked the referral because when they see that things are not going well or what you expect will not happen, you give you some other chances to save the life of the baby and yours, and also now I have my baby even though I have a wound.”

**Interviewer: Q8 What did you not like about being referred?**

**Respondent 2:** “during referral, aah I was not feeling very well at that time because when they told me you go to RRH, aah I didn’t expect that to happen(pause) I thought I was going to try to give birth normally but the baby refused to come. And also, ‘they told me that the doctor who works in the theatre was not present, I would have had my operation there’.”

**Interviewer: Q9 What has been your overall experience when you were referred?**

**Respondent 2:** “I can say my overall experience was good because I moved with three doctors in the ambulance and also the driver was good, I realized that I was care for and this gave me hope for a good outcome

**Interviewer: Q10 What was your experience on the road during referral?**

**Respondent 2:** “our driver was slow and simple and careful because he knows that he is taking patients and pregnant mothers who are already in pain, so he was driving slowly and careful to avoid accidents so as to reach safely. We also had three doctors in the ambulance with us you kept on comforting and giving hope to me. We reached safely even though it was at night”

**Interviewer: Q11 What was your experience when you were received here? What was the quality of care you received here?**

**Respondent 2:** “when we reached here at around midnight, they received me immediately and examined me and they said my Uterus is at 6 cm. They repeated the examinations until the final doctor came and examined me at around 3 pm and they told me that ‘the baby was big, and I needed to have to go to theatre for operation.’ All that time I was moving around thinking the baby might go down as expected but it did not”

**Interviewer: Q12 how were you handled when you arrived here in RRH?**

**Respondent 2:** “the care was immediate, yes immediate. We opened up the file and they started working on us. They took me to the labor room for some examination at that’s where they told me that my uterus is around 6 cm. that was around 1 am. Then they filled the file and told me the next examination will be t 5 am but instead they repeated at 9 am. Then they check me again at around 11 am. I waited till 3 pm when they told me that the baby was big and I was not able to deliver since I had a previous scar.”

**INTERVIEW DETAILS:**

**Interview time:** 15 minutes:22 seconds

**Interview location:** Postnatal ward (.......)

**Moderation language:** English

**Participant number:** 03

**Conversation language definitions:**

**Bold:** Title/interview questions

**Normal text:** Respondent

**Silent talk:** (Pause)

**I:** Interviewer

**R:** Respondent/participant

**Q:** Interview question

| Demographic data: | Response |
| --- | --- |
| Initials  Age:  Marital status: Married  Education level  Parity  Source of referral  Contacted the receiving facility prior to referral  Mode of transport  Distance from the referring facility  Mode of delivery  Outcome of baby after delivery | KS  27 years  Married  P.7  3  HC-IV  Yes  Taxi  25 km  Caesarean section  Alive with the mother |

**RECORDED INTERVIEW**

**Interviewer: Q1 Tell me about your delivery?**

**Respondent 3:** “My labor started at around 1pm. I stayed at home, hoo-ome. In the morning there was no any vehicle to go to the hospital because I stay very far from …. because I was in the village. Finally, we got a vehicle and we reached health centre -IV at around noon of the following day. So, when we reached there, the nurses were there, they welcomed me very well, they checked me. When they checked me one nurse said she did not see the head of the baby and was very far maybe you are still some hours. So, I waited again for the next check, time reached and the said you will not push the baby because the head is not here, the head is still very far and now your placenta is near the way. Then she told me ‘Now we are going to transfer you to RRH’. At that time, I did not have any transport money with me, so I called my husband at 11 am to come. When he came at around 1 pm we got a taxi to come because the ambulance was not there, they told us to wait that the ambulance is coming for us, I said aah, ambulance is going to delay so we left and reached here vey well. They welcomed me. The nurses came and checked me and they said I was 7 cm and they told me to walk-walk around and take sweet tea. I tried to walk then around 9 pm they checked me and they said the baby was still at 7 cm. so at that time they called another nurse, when she came, she asked me if they operated me? I told her yes, they operated me my first born, the second one I produced. So, she said aah, you will produce, let us give you 3 hours. So, when it reached 3 hours, the water came then they took me again at 11 pm when it was about to reach midnight. So, when they checked me again, they said aah the baby is still in 7 cm. they said you bring the things we are taking you now to the theatre. When I reached theatre, they handled me very well until I came out

**Interviewer: Q2 Tell me about your experiences when you were referred?**

**Respondent 3:** “my experience was not very bad, the taxi brought me up to inside the hospital then I moved here to the ward. By then I was feeling that am going to lose my life and the baby. While in the taxi they gave me and my husband the front sit since I was in pain.”

**Interviewer: Q3 When you were told, you are coming to RRH, did you have any reservations/reluctance on referral? What were your thoughts about referral?**

**Respondent 3:** “when they told me they are transferring me to RRH, I was willing to come because now the baby was not coming but have labored long and still at 7 cm this was not good and I began to worry that I may lose my life and the baby as well, I said all children are not the same, my second born I was able to produce normally.”

**Interviewer: Q4 What do you think about the care of mothers from where you were referred? Would you rate the quality of care where you were referred?**

**Respondent 3:** “the care of mothers at ….? For me I don’t know because my first two children I delivered at Hoima when I was living with my husband at ........ But when I was taking drugs for maternity at …. they told me when the time comes, don’t deliver at home you go to the health centre, so when they say you go to main hospital you go. So, I don’t know much at… very well, I cannot lie you. But when I arrived at …. and they checked on me they told me not to push or do anything just be until we transfer you.”

**Interviewer: Q5 What do you think about the quality of care you have received from here?**

**Respondent 3:** “the care? The care here is good, is not bad. I got treatment every time very well, they attended to me and monitored me any time I report pain they give me treatment and also the hygiene here is very good, also the nurses tried to give me time to deliver but the baby remained at 7 cm then they just had to take me for operation.”

**Interviewer: Q6 Was this the place you were referred to?**

**Respondent 3:** “yes this was the place. When the doctor told me I transfer you to RRH, I asked her why don’t you transfer me to …HC-IV? She told me that if you want you can go but she said that she has already called RRH that you are coming. I said I will not go to …. or …., let me go to RRH”

**Interviewer: Q7 What did you like about referral?**

**Respondent 3:** “I have liked referral because I am still alive and my baby is alive too. I also liked the nurses they gave me good advice to come to RRH and they even made a call to inform them that am coming. I have also liked everything what is going on here at the main hospital.”

**Interviewer: Q8 What did you not like about being referred?**

**Respondent 3:** “I did not see anything bad during referral except that the ambulance had gone somewhere so I could not wait for it to come I had to use a taxi so that I don’t delay waiting when am not sure the time it will arrive.”

**Interviewer: Q9 What has been your overall experience when you were referred?**

**Respondent 3: “**My experience has been good because the doctors advised me well about why I have to go to main hospital and also they made calls to make sure I was going to be attended here when I arrive so they care for me well.”

**Interviewer: Q10 What was your experience on the road during referral?**

**Respondent 3:** “on the road they gave me and my husband the front seat so I did not feel much being in pain since the drive knew I was in pain and he had to drive slowly on he humps also because the front has little effect of the humps.”

**Interviewer: Q11 What was your experience when you were received here? What was the quality of care you received here?**

**Respondent 3:** “the care here is very good they attended to me very fast when I arrived because the nurses were there, I had to open a file and they checked on me. they also kept on checking on me to make sure the baby was fine and they did not hurry to take me to theatre, they gave me some time but it did not help then I ended up in theatre.”

**Interviewer: Q12 how were you handled when you arrived here in RRH?**

**Respondent 3:** “They handled me very well because I reached like this, they take me inside then they checked me. It took like about 30 minutes for them to check me because I had to open a file and they were asking me about the labor. In the theatre they handled me very well, because when I reached, there they had organized things very well there so they started working on me. It took like 40 minutes when the baby is out”

**INTERVIEW DETAILS:**

**Interview time:** 15 minutes:52 seconds

**Interview location:** Postnatal ward (RRH)

**Moderation language:** English

**Participant number:** 04

**Conversation language definitions:**

**Bold:** Title/interview questions

**Normal text:** Respondent

**Silent talk:** (Pause)

**I:** Interviewer

**R:** Respondent/participant

**Q:** Interview question

| Demographic data: | Response |
| --- | --- |
| Initials  Age:  Marital status: Married  Education level  Parity  Source of referral  Contacted the receiving facility prior to referral  Mode of transport  Distance from the referring facility  Mode of delivery  Outcome of baby after delivery | TA  29 years  Married  Tertiary  3  HC-III -  Yes  Ambulance  6 then 48 km  Caesarean section  Alive with the mother |

**RECORDED INTERVIEW**

**Interviewer: Q1 Tell me about your delivery?**

**Respondent 4:** “My delivery? This is the 3^rd^ child. My first child was a caesarean section and the 2^nd^ as well. So, when I started experiencing contractions, I went to the health centre at HC-III where the nurse handled me very well and she was like I want to give you a chance so that you push and I was ready for that. She treated me in the morning and told me to come back in the afternoon. I went but i found myself was still at 2 cm then she was like am not going to work at night duty but the nurse who will work on night duty will attend to you when I reached there the nurse was like you have to be referred, so we had to be referred, they called the ambulance and they asked some little money for transport then we went to … main hospital. When we reached there, the doctor was there but the challenge we got was they never had medicine in the theatre so they told me we have to refer you again. Because I was in too much pain, I was like am ready to be referred.”

**Interviewer: Q2 Tell me about your experiences when you were referred?**

**Respondent 4:** “When I was referred, I was like you know we consider God most of the times, I was like let God take his charge and control. But when I heard it was RRH, I was confident I knew all things might go well because it is big hospital. That was the thing I had experienced some years back; I had not been t ....... before.”

**Interviewer: Q3 When you were told, you are coming to RRH, did you have any reservations/reluctance on referral? What were your thoughts about referral?**

**Respondent 4:** “I knew that I can’t push as a mother because even my first bon, I didn’t even get these contractions, the 2^nd^ born I had contractions but I failed because the cervix stopped at 2 cm, it did not open. So, this one also they tried to test me, I was like it is 4 cm aah! I had that expectation in my mind that it will be a C/section again. The fears I had came because of pain but there were no fears at all.”

“When I was told about RRH, I had the confidence because I knew it was a big hospital though I had not been here before I knew things will be alright for me and baby so I had to accept because there was no option now even no money for operation at ….. which was near so I placed everything to God to take charge and control. With the past experience of the operations, I had no fears.”

**Interviewer: Q4 What do you think about the care of mothers from where you were referred? Would you rate the quality of care where you were referred?**

**Respondent 4:** “At …HC-III they were the best because they had to make all things so that I may reach … general Hospital. We had three nurses from …. who escorted me and my attendant in a tricycle motorbike ambulance to ........ At …. general hospital, the care was good because as I reached there, the doctor had to come immediately to check and was like you are at 4 cm but you need to be transferred to …. because the medicine is not there in the theatre and another thing you have two (2) previous scars, so you are a risk mother. Then the nurses started calling. They called here, and they were like we are free, you bring the patient. Then they said do you have something like transport because we have to pour fuel in this car the ambulance because it is empty. I had the transport at that time, I gave them 30,000/= they had asked for then we set off from ....... to ....... by ambulance.”

**Interviewer: Q5 What do you think about the quality of care you have received from here?**

**Respondent 4:** “When I reached here, the care was not also bad, I came in the night and the nurses were there. They welcomed me and they checked the notes and were like aah! Why are you a two-scar mother and you risk yourself? I was like that was the information or the advice from the nurse, they had to arrange very first and take me to the theatre.”

**Interviewer: Q6 Was this the place you were referred to?**

**Respondent 4:** “from ....... yes. Before that I was first referred from …..health centre-III to …. general hospital a distance of about 6.2 km from …. general hospital. Unfortunately, when we arrived at there, they said there was medicine in the theatre then they gave me two options; you go to ….. in ....... or RRH? When I inquired about the price in …., they told me 800,000/= for operation which I could not manage so I was like let me use RRH a distance of 46 km because it is a government hospital.”

**Interviewer: Q7 What did you like about referral?**

**Respondent 4:** “For sure when I reached here, the care was nice. I have like referral because it saved my life and that of my baby and also the way the nurses comforted me during the way to make sure that my pain was minimal and they kept on encouraging me to take heart. In general, I have like referral because they take care of the patient”

**Interviewer: Q8 What did you not like about being referred?**

**Respondent 4:** “what I didn’t like about being referred is that we got some challenges because when you reach here, some things are bought, like some medicines, they told us to buy jik, Omo for the theatre, the kavera (Polythene sheet) to use. Those are the challenges.”

**Interviewer: Q9 What has been your overall experience when you were referred?**

**Respondent 4:** “My overall experience on referral is good because when they sent me from …. HC-III to main hospital, at main hospital the medicine was not there and they had to say go to …. However, I got what I wanted (pause), I got what I wanted. I got treatment. As for now I say am free and am well. If I was to remain at …., I don’t think I would have made it. But when they referred me here, I have seen and I have got what I wanted.”

**Interviewer: Q10 What was your experience on the road during referral?**

**Respondent 4:** “For sure the pain was too much that the two nurses sat behind me to give support when the pain was coming, those nurses have to hold me, and they had to tell me to take heart we are almost reaching. But I was in too much pain and the baby was not going down. (Pause) the road was humpy from ....... to ......., it was not congested because it was at night, the traffic was not too much. But humps? I experienced the challenge of humps when the car I humping I felt a lot of pain.”

**Interviewer: Q11 What was your experience when you were received here? What was the quality of care you received here?**

**Respondent 4:** “The care is Soo nice because when you call like Musawo I need this, the musawo is ready for you all the time. There are also these students they even help us when the Basawo’s are also busy, these students’ come in. when you need assistance, they are always there, you have someone at least to call all when in need and they respond fast. The care here is Soo nice!”

**Interviewer: Q12 how were you handled when you arrived here in ....... Regional Referral Hospital?**

**Respondent 4:** “The handling was not bad, we arrived here at around 1:40 am were they received me and they told me to buy the file and I was ready for that and paid, they did everything, they received me very well. They first took me to the bed for test, at that time, I was pouring water as if the baby was almost coming. So, they had to say ‘aah, you come and we go to the theatre because we can’t risk you’. from the time I arrived it took like 1 hour for them to take me to the theatre because they were still organizing some things like putting cannula, catheter, calling the doctor and theatre, and removing blood.”

**INTERVIEW DETAILS:**

**Interview time:** 14 minutes:18 seconds

**Interview location:** Postnatal ward (.......)

**Moderation language:** English

**Participant number:** 05

**Conversation language definitions:**

**Bold:** Title/interview questions

**Normal text:** Respondent

**Silence: (pause)**

**I:** Interviewer

**R:** Respondent/participant

**Q:** Interview question

| Demographic data: | Response |
| --- | --- |
| Initials  Age:  Marital status: Married  Education level  Parity  Source of referral  Contacted the receiving facility prior to referral  Mode of transport  Distance from the referring facility  Mode of delivery  Outcome of baby after delivery | NE  27 years  Married  S.3  5  … HC-III  NO  Boda- boda  16.2 km  Caesarean section  Alive with the mother |

**RECORDED INTERVIEW**

**Interviewer: Q1 Tell me about your delivery?**

**Respondent 5:** “it was at around 11 am I was going to the market, I bathed very well, I went to the market, I reached on the way then the stomach started paining me. I said what is this, I knew it was now time, I went back home, I sat there, I cooked food, gave my babies then waited for my husband to come then we came to hospital (HC-III). I reached there they examined me and they told me aah, the way is not open, now they said you move around. I moved, I moved, I moved. Then at 6pm they examined me again and they said, the way the at 3 cm. They said go move and take tea. They kept me the whole night while checking and at 8 am they checked me I was still at 3 cm. I moved and moved then at 1 pm the examined me and it has not expanded. Then I said aah! What is the problem? they said ‘the baby is very big, is fat now it comes slowly’. I sat there till 5pm they checked me and they said, aa, aah! There is no change, I said you transfer me, they said ‘let us wait’. Then when it reached 4 am I saw the stomach was very tough and paining I said I am not going to reach the morning then they gave us transfer. We reached here at around 4 am in the morning, they examined me and they said ‘you are going to the theatre straight way’”.

**Interviewer: Q2 Tell me about your experiences when you were referred?**

**Respondent 5:** “I was very bad, very bad, I was feeling bad and if I was to continue there for another 2 hours, I could have died because I was feeling very bad and I was the one who said aah you refer me because those nurses were delaying and I was feeling the stomach was very tough and hard.”

**Interviewer: Q3 When you were told, you are coming to RRH, did you have any reservations/reluctance on referral? What were your thoughts about referral?**

**Respondent 5:** “I was the one who decided for transfer but to her she said ‘if you reach in the morning, I will transfer you’, but I said, when will morning reach? and I said let me go. I called my husband because he had gone to sleep with the little babies at home. I told him that have been referred you come with the Boda-boda. There are some boda-bodas in the village then we prepared and came here. Although I had fears, I was feeling very bad, I said aah, am I going to die? That was the first thing that came to my mind I was not feeling good because it was my first time to have operation. All the four children I was pushing them but this one I was feeling very bad and the stomach was very tough so I had to request for a transfer though I had fear for operation.”

**Interviewer: Q4 What do you think about the care of mothers from where you were referred? Would you rate the quality of care where you were referred?**

**Respondent 5:** “I can say with my experience, the care is fair because they kept monitoring me and they delayed to transfer me until I insisted when the stomach was tough and hard and I was feeling very bad. If I had not asked for transfer then I would have lost my baby and my life”

**Interviewer: Q5 What do you think about the quality of care you have received from here?**

**Respondent 5:** “the care is very good because yesterday, I was feeling very bad because of the pain they treated me and now am somehow improving and not feeling very bad like yesterday. The treatment is also there except some they told me to buy because they were not there.”

**Interviewer: Q6 Was this the place you were referred to?**

**Respondent 5:** “it was my decision and my choice. The nurses had not made a decision to referred me until I insisted because ....... is a big hospital and I knew I was going to be helped.”

**Interviewer: Q7 What did you like about referral?**

**Respondent 5:** “I have like referral because when I reached here, they saw my condition was very bad, they said let us first work on this one, let the others wait because they are not very bad that is why I have liked referral. I have also like treatment, they treated me very well. If you call any nurse or doctor, they come very fast and attend to you.”

**Interviewer: Q8 What did you not like about being referred?**

**Respondent 5:** “actually to me everything was good and I did not see anything bad that I did not like, may be because my condition was very bad and I was almost dying and so everybody was concern.”

**Interviewer: Q9 What has been your overall experience when you were referred?**

**Respondent 5:** “my overall experience was very good because I took decision to myself to ask for transfer and when I reached here, they worked on me very fast since my condition was very bad. So, it was a very good experience.”

**Interviewer: Q10 What was your experience on the road during referral?**

**Respondent 5:** “on the way I experienced a lot of pain because I used a Boda-boda and when it reaches the humps aah, you feel very bad the pain increases. The way was clear at night and we reached safely.”

**Interviewer: Q11 What was your experience when you were received here? What was the quality of care you received here?**

**Respondent 5:** “it was very good because they worked on me very fast as they saw that my condition was very bad and they had to take me first to theatre immediately and I had my baby. The care here is very good because nurses and doctors are concern, they kept checking and asking how you are feeling and they respond when you call for help.”

**Interviewer: Q12 how were you handled when you arrived here in RRH?**

**Respondent 5:** “as I had told you they handled me very well. When I reached like this, they checked me and they said this one is very bad let us work on her first. So, they sent my husband to buy some things and I entered the theatre at around 4 am coming to 5 am and they removed me at 6 am in the morning.”

**INTERVIEW DETAILS:**

**Interview time:** 17 minutes:11 seconds

**Interview location:** Postnatal ward (.......)

**Moderation language:** English

**Participant number:** 06

**Conversation language definitions:**

**Bold:** Title/interview questions

**Normal text:** Respondent

**Silent talk:** (Pause)

**I:** Interviewer

**R:** Respondent/participant

**Q:** Interview question

| Demographic data: | Response |
| --- | --- |
| Initials  Age:  Marital status: Married  Education level  Parity  Source of referral  Contacted the receiving facility prior to referral  Mode of transport  Distance from the referring facility  Mode of delivery  Outcome of baby after delivery | MA  22 years  Married  Tertiary  1  HC-III – - .......  Yes  Boda-boda  16.2 then 23 Km  Caesarean section  Alive with the mother |

**RECORDED INTERVIEW**

**Interviewer: Q1 Tell me about your delivery?**

**Respondent 6:** “I started one week ago experiencing little pain and when it increased, I went to ….HC-III at night at around 10 pm. On reaching, I found there the midwife she did examination and was like am not able to push the baby and I have to refer you to Regional referral hospital. She did not even tell me the findings of the examination so that I may know, only that she was my pelvis is not all that wide to allow the baby to pass through. But before I went to the health centre, 2 weeks back I was experiencing some little pain when it became severe, I had to go to …health Centre-III where they referred me to ........ But I insisted and went home until when the pain became much.”

**Interviewer: Q2 Tell me about your experiences when you were referred?**

**Respondent 6:** “it was automatic I thought of a C/Section because I refused to come, the very day they referred me thinking things will be fine but instead there was not change.”

**Interviewer: Q3 When you were told, you are coming to ......., did you have any reservations/reluctance on referral? What were your thoughts about referral?**

**Respondent 6:** “I got anxiety because it was even my first time to enter there. I got that anxiety because that very day they referred me I had to reach here, I insisted but said, for me I have to make it I have to push me baby, I had to go back home. Two days after, the pain was severe so I had to come back straight here. I had to use private means to come here.”

**Interviewer: Q4 What do you think about the care of mothers from where you were referred? Would you rate the quality of care where you were referred?**

**Respondent 6:** “what I know there because it is a government health facility, when you go there to deliver, they give you a free Mama kit with all the properties, then if you are not yet the examine you and after they ambulate you. If you have not yet reached the time, then when you are ready, they take you to the labor suite then they work on you. The staff are co-operative and they are free, after delivery, they health educate you on what to do normally they give us health talks after delivery, they tell you be washing the baby, clean yourself.”

**Interviewer: Q5 What do you think about the quality of care you have received from here?**

**Respondent 6:** “to me, I think the care here is for the basawo to tell us what to do because us the mothers we come when we don’t know anything so we expect health talks like after coming from theatre, you need to eat at this time, the pain at this time, and monitoring us. Even in treatment, you are giving us medicine, like for us who go there in the theatre, they give us prophylaxis for the wound you again give us another to ensure that we do not get other infections. the services generally are good. Ate, they are good at everything they do to you. They first explain the services before they give you. They first explain the services, and the good thing the doctors do not do anything on you before explaining and you really understand. Like the other time when I failed to consent, they had to counsel me and I become cooperative, so they did not force me.”

**Interviewer: Q6 Was this the place you were referred to?**

**Respondent 6:** “yes, I chose ....... because those people had told me to come to ....... because me who doesn’t know there, I have to go by what the doctor or the midwife tells me and advised me, so me I also accepted.”

**Interviewer: Q7 What did you like about referral?**

**Respondent 6:** “I have like referral because they give you information and then you make a decision and also when they tell you to go where they recommend, they know why and you have to be ready. But also, I had to make my own decision later. I have liked the care given here since I came, the health workers are really concern about their mothers in the ward.”

**Interviewer: Q8 What did you not like about being referred?**

**Respondent 6:** “me I cannot say that I don’t like this because all the services I was thinking of the basawo’s gave me.”

**Interviewer: Q9 What has been your overall experience when you were referred?**

**Respondent 6: “**my overall experience has been good because finally I came to understand that the basawo was right to refer me in the first place but me I thought I was able to manage to push not until things did not work the way I wanted. So, I commend them for the knowledge they have in helping mothers who are difficult like me.”

**Interviewer: Q10 What was your experience on the road during referral?**

**Respondent 6:** “I experienced a lot of pain on the way because the humps aah! If you reach on the humps, you feel very bad, the pain increases.

**Interviewer: Q11 What was your experience when you were received here? What was the quality of care you received here?**

**Respondent 6:** “I say it was good other health workers when you refuse their services and then you come back, they will just ignore you, but when I came back, they had to welcome me and they did a re-admission so I would say it was good.”

**Interviewer: Q12 how were you handled when you arrived here in Regional Referral Hospital?**

**Respondent 6:** “I was welcomed, they had to show me where the pregnant mothers go, they were there to give me the services I needed. They attended to me immediately. They were like if you have not done a scan, they had to remove the sample from me they took to the lab. They gave me enough fluids because they were preparing me to go to theatre. They told me the baby had a tight cord around the neck twice, then they told me also my OS is closed ate, I was feeling pain!”

**INTERVIEW DETAILS:**

**Interview time:** 17 minutes:15 seconds

**Interview location:** Postnatal ward (.......)

**Moderation language:** English

**Participant number:** 07

**Conversation language definitions:**

**Bold:** Title/interview questions

**Normal text:** Respondent

**Silent talk:** (Pause)

**I:** Interviewer

**R:** Respondent/participant

**Q:** Interview question

| Demographic data: | Response |
| --- | --- |
| Initials  Age:  Marital status: Married  Education level  Parity  Source of referral  Contacted the receiving facility prior to referral  Mode of transport  Distance from the referring facility  Mode of delivery  Outcome of baby after delivery | NG  32 years  Married  S.2  3  ….HC-III  Yes  Ambulance  17.3 Km  Caesarean section  Alive with the mother |

**RECORDED INTERVIEW**

**Interviewer: Q1 Tell me about your delivery?**

**Respondent 7:** “when I went to ......., I reached there at around 8 in the morning because the pain started in the night yesterday. They received me and they checked on me and then they told me, ‘I cannot deliver, when I deliver, I may bleed a lot then they called the ambulance that very time and referred me to come to ........ They told me don’t even remain you just go because of your condition. When the ambulance they asked us for transport to bring us to ........ We were two mothers referred so each of us paid 40,000/= each. When we reached ......., they checked on me and some were saying she can deliver and some were saying she cannot deliver. so, I just remained there and they told me you first wait. I waited then they decided to operate me.”

**Interviewer: Q2 Tell me about your experiences when you were referred?**

**Respondent:** “I felt now (paused) that they are going to operate on me because they told me if I deliver, I might bleed until death so I was fearing that. By that time, I was not having the money for transport and for food while here so my husband had to go back home to look for the money and he came back after two hours from the time he left for home.”

**Interviewer: Q3 When you were told, you are coming to ......., did you have any reservations/reluctance on referral? What were your thoughts about referral?**

**Respondent 7:** “I was willing to come because they had already told me that ‘if I deliver, I will bleed to death.’ So, it is better to come to a big hospital where they can operate me and even give blood if I bleed a lot.”

**Interviewer: Q4 What do you think about the care of mothers from where you were referred? Would you rate the quality of care where you were referred?**

**Respondent 7:** “the care from there, some of the staff are talking well but some hmm! they are not because when I was still going for maternity, I told them the problem that I was having, they told me we can’t help you, you go to .......’s Hospital (some clinic there in .......). They refused to give me treatment, or advice instead to go to .......’s Hospital. So, I had to go to another clinic not that one they had told me. They worked on me but it did not even improve, the problem just remained like that till now after spending the money. Therefore, I say the care is fair (Paused) because they accepted to escort us in the ambulance.”

**Interviewer: Q5 What do you think about the quality of care you have received from here?**

**Respondent 7:** “the care here is good because they are caring for me and I also see them caring for other mothers equally. The nurses and doctors talk very well. For treatment, they give us but some they say by from outside then they write a small paper to go and buy.”

**Interviewer: Q6 Was this the place you were referred to?**

**Respondent 7:** “yeah this was the very place I was referred because of what I told you about my condition which normally comes when I am pregnant and they told me it needs a big hospital to deliver or else I may bleed until death.”

**Interviewer: Q7 What did you like about referral?**

**Respondent 7:** “I liked referral because they have saved me and also the midwife who escorted us was concern about us in the ambulance. Now I am recovering from the operation. I have also liked their management in general the staff, the ward, the cleaning, all.”

**Interviewer: Q8 What did you not like about being referred?**

**Respondent 7:** “the transport cost is what I did not like about referral because my husband had to go look for the money and it took him 2 hours to return back from home or wherever he went to look for the money.”

**Interviewer: Q9 What has been your overall experience when you were referred?**

**Respondent 7:** “my overall experience has been good because they were able to save my life and now am recovering from the operation and the baby is fine too. They first gave me time as they were deciding to operate me and if that the baby was not up, I would have pushed.”

**Interviewer: Q10 What was your experience on the road during referral?**

**Respondent 7:** “there was no problem on the road. We had a midwife who escorted us to ....... regional hospital. The road was good and we reached safely.”

**Interviewer: Q11 What was your experience when you were received here? What was the quality of care you received here?**

**Respondent 7:** “When we reached ......., they checked on me and some were saying she can deliver and some were saying she cannot deliver. so, I was just there thinking now what is this and they told me you first wait. I waited then I think they made a conclusion and decided to operate me since that condition the one I told you comes when I am pregnant could not allow the baby to pass through.”

**Interviewer: Q12 how were you handled when you arrived here in ....... Regional Referral Hospital?**

**Respondent 7:** “They received me well, the midwife who escorted me explained to them then they gave me a bed. They started working on me by checking then after they told me ‘She can push the baby;’ they then told me to ‘start moving around and take tea.’ When they checked me again, they said ‘the baby was still up’ then they told me ‘We go for operation’, that is how I ended up in the theatre.”

**INTERVIEW DETAILS:**

**Interview time:** 21 minutes:23 seconds

**Interview location:** Postnatal ward (.......)

**Moderation language:** English

**Participant number:** 08

**Conversation language definitions:**

**Bold:** Title/interview questions

**Normal text:** Respondent

**Silent talk** (Pause)

**I:** Interviewer

**R:** Respondent/participant

**Q:** Interview question

| Demographic data: | Response |
| --- | --- |
| Initials  Age:  Marital status: Married  Education level  Parity  Source of referral  Contacted the receiving facility prior to referral  Mode of transport  Distance from the referring facility  Mode of delivery  Outcome of baby after delivery | NJ  35 years  Married  S.3  5  ….HC-IV  Yes  Private car (Hired)  20.8 km  Caesarean section  Alive with the mother |

**RECORDED INTERVIEW**

**Interviewer: Q1 Tell me about your delivery?**

**Respondent 8:** “My labor started on the 30^th^ October 2023 where I felt pain then I remained at home the second day was 31^st^ October I also felt pain and it was going on and off, then on 1^st^ November 2023, the pain increased and I was feeling badly off. I said no let me go to the hospital. So, I went to the hospital at around 10 am I reached there and they checked on me then reaching at 2pm they again checked on me and they told me that you move in this compound. After moving, I moved it reached at 9 pm they checked on me and told me not to push because we have seen your vagina is now becoming oba what? (swollen) inside there don’t push. Then also they said that your pressure in high they told me to go and buy tablets, I went to the shop and bought then they told me to take one and one after another 30 minutes. Reaching at 10 pm they checked me and they said your temperature is high now we will not manage for you to produce from here, for us here at this time doctors are not here, instead of being here we are sending you to ....... regional hospital. I told them you help me at this time I don’t have money for going there. They told me that there is no way of helping you because for us here the doctors here work at day time and now it is night, we can’t get the doctor let us call the doctor from ....... if it is working you go there. Now the nurse called the doctor from ........ The doctor said why can’t you work on that guy? They said the doctor who was to work on her is in ........ Now they wrote for me a transfer letter and they said you go and buy a canular and a catheter we put for you so that when you reach there, they work on you very fast. Then I also said let me go and buy. I was having my four friends with me two ladies and two men (one my husband). Now my husband came and asked me what is wrong? I told him they have sent me to ........ The husband said now I don’t have money, what can we do? He said you first be patient I go to my boss if he can help me. I was still at the health centre. Now he went, talked to the boss and the boss said, now if they told you to go to ......., there is no way, now they have already finished to write the transfer. He said let me look for transport, are you going to use a boda-boda? Then my husband said she cannot be able to sit on the boda-boda because we are four. Then he went to the stage of .......then one of the four friends of mine said his friend has a car let us go to the garage then he told us he wants 80,000/= then my husband said right now I don’t have 80,000/=, but am requesting you help us I have 50,000/=, you take her they work on this woman then the balance of 30,000/= I will pay you after coming back. The husband gave the man the money, goes to shell, takes mafuta (fuel) then he came to pick me with my friends. We reached here at around 11 pm. They took me to the nurse, the nurse checked on me and she said what is in the file is not resembling what they told me, the file says your vagina is now becoming big (swollen) and coming out and if it come out you will not manage to push the baby, one or two of you will be off unless you go to the doctor’

The nurse here said ‘those people of .......we normally get the same salary with them, now they are sending this way people, this one is not the first one. There is another one again they had sent when they had told her other words yet in the file, they had written another word.’

Now they said you go to that bed and we check on you. But before she first quarreled about .......staff then she told me to go to that bed and I check on you. She checked and said the pressure is high but inside it was 8 cm the baby was not coming down; it was going up. She called another doctor it was a man she said that man give that girl one tablet she put down under the tongue but don’t use water, just put there and keep quiet. I was feeling pain and I also said, musawo help me-e, am badly off even though I don’t have money I will pay you before I go, she said quiet, you do what I have said.

As I kept quiet, I found four women one in theatre and three were still waiting but they said let us wait for that one to be taken and we remained two. So, they decided to say to that ‘woman you wait we are taking this one first because she is badly off, we have seen the result is bad.’ They said ‘do you have money? We want 15,000/=’, I said right now I don’t have but I will pay you work on me they said ‘you sign,’ very first I signed very quickly then they told me to go inside with another guy (student). We entered there and they worked on me very quickly till I came out. They didn’t take long and the baby was out.”

**Interviewer: Q2 Tell me about your experiences when you were referred?**

**Respondent 8:** “At …., they told me if you go to town those people they don’t care of patients, but for me I was badly off I said let me go and try my luck! I told my husband let us go, God knows my problems. I came they worked on me and now I am happy. I got something different from what they had told me from .......about here.”

**Interviewer: Q3 When you were told, you are coming to ......., did you have any reservations/reluctance on referral? What were your thoughts about referral?**

**Respondent 8:** “me, I was willing and open because I was badly off, I said let me even sign from here before taking me there, I was feeling pain, a real pain.”

**Interviewer: Q4 What do you think about the care of mothers from where you were referred? Would you rate the quality of care where you were referred?**

**Respondent 8:** “Those mothers are I need of money, they are not at work, when you have money, they work on you very fast, but when you don’t have money, they leave you there. But when you have money, they say let us write for you this medicine you go and buy. The other thing is immediately you reach there like this! They say give the money for gloves, when you don’t have, they don’t work on you. So, because of that I can give them a rate of poor because they did not help me.”

**Interviewer: Q5 What do you think about the quality of care you have received from here?**

**Respondent 8:** “the care is good because at that time I was not having money but they worked on me and I did not even pay anything like we used to there.”

**Interviewer: Q6 Was this the place you were referred to?**

**Respondent 8:** “yes this is the place I was referred; they did not tell me of any other hospital. At first, I did not like to come but as they insisted that they cannot work on me, I accepted to come”

**Interviewer: Q7 What did you like about referral?**

**Respondent 8:** “Here in ....... referral Hospital, I have liked the care, the care is good because they care for me since it was my first time to come here. The rest of my children I used to deliver from ....... (….HC-IV).”

**Interviewer: Q8 What did you not like about being referred?**

**Respondent 8:** “Anti I told you I used a hired car so it was expensive for us since we have a debt to pay back so I did not like that about referral. And also, I did not like the way the nurses tried to discourage me from coming here because I felt they just needed money,”

**Interviewer: Q9 What has been your overall experience when you were referred?**

**Respondent 8:** “it was good because what I wanted, I got. Time reached for theatre also they were ready and they worked on me, but from there, they told me doctor is not there more over it is called health centre four! Doctor is supposed to be there.”

**Interviewer: Q10 What was your experience on the road during referral?**

**Respondent 8:** “My experience on the road was not bad because the driver had no way of disturbing us on the way, we came when he knew that I am carrying a pregnant mother who is in pain and he was driving well. Reaching here he even helped me to enter inside the labor ward, then he left after I entered.”

**Interviewer: Q11 What was your experience when you were received here? What was the quality of care you received here?**

**Respondent 8:** “Here in ....... referral Hospital, the care is good because they cared for me since it was my first time to come here. I came with that mind of these people don’t care but I was surprised they care was not what they told me. They don’t even wait as soon as they condition is bad; they work on you very fast and they also explain to other mothers why they have to wait.”

**Interviewer: Q12 how were you handled when you arrived here in ....... Regional Referral Hospital?**

**Respondent 8:** “At first as if they were quarreling (complaining), in fact they quarreled, that ‘how can you send someone when she is badly off like this and those people also get salary like us, there is no difference in salary, now how can a patient come and you don’t work on them then you send for us and they know that this way we have many patients, those people are not doing good, they are doing bad. After complaining and arguing among themselves, she said go up and I check on you and also follow me where I am going. I also woke up and followed as she told me, and that the noise should stop there, I also kept quiet, followed the musawo and I got what I wanted in fact she was not happy with the people who referred me, I think she was disgusted but at least she helped me.” For the treatment, they were writing for me medicine, I also went out to buy (Pause) but some they are giving us for free and those they don’t have they send us to buy. I see the cannulas and the drip sets they are giving us for free but there everything is money.”

**INTERVIEW DETAILS:**

**Interview time:** 17 minutes:20 seconds

**Interview location:** Postnatal ward (.......)

**Moderation language:** English

**Participant number:** 09

**Conversation language definitions:**

**Bold:** Title/interview questions

**Normal text:** Respondent

**Silent talk:** (Pause)

**I:** Interviewer

**R:** Respondent/participant

**Q:** Interview question

| Demographic data: | Response |
| --- | --- |
| Initials  Age:  Marital status: Married  Education level  Parity  Source of referral  Contacted the receiving facility prior to referral  Mode of transport  Distance from the referring facility  Mode of delivery  Outcome of baby after delivery | NG  16 years  Married  S.1  1  ....... HC-III  Yes  Boda-boda (Motorbike)  28 km  Caesarean section  Alive with the mother |

**RECORDED INTERVIEW**

**Interviewer: Q1 Tell me about your delivery?**

**Respondent 9:** “I went to maternity on Wednesday. When I reached there, they told me I had malaria, ‘you go to the laboratory and check for the test’ then they found that there is no malaria. They took me to another room to check if am near to go into labor. I went to the labor when some water is coming out, they told me that ‘don’t go back home, you remain here, let them go and bring you some clothes, mama kit, and baby clothes. Time reached for checking and they told ‘you might deliver at 3pm’. It reached 3pm and they told me ‘The baby is at 6 cm, my might deliver at night’. It reached night and they check me and it was still at 6 cm. Then they told me ‘You might have some problem from where the baby is coming from’ they gave me some injection and the stomach stopped paining me. They told me to go back home and come tomorrow. I said let me sleep I will go tomorrow, then we slept. In the morning I finished to get injection and I went home. When I reached home, the stomach starting paining, water was still coming out, then we went back when the stomach is still paining me. They checked and they said ‘you might deliver at 12pm’, that was on Thursday. It reached 12pm and they checked me and they said it was still at 6 cm and they told me you might deliver at 6 in the morning. They checked me and it was at 6 cm and they said let us wait for 1pm. It reached 1pm and still it was at 6 cm then they told me let us transfer you to .......or ....... Hospital’. I told them that my father works at ......., you transfer me to ........ That doctor made a call to sister then we came this way on Thursday. Then it reached Friday no baby, it reached Saturday and then they told me ‘You might go for operation’ aaya even me I got interested, let me go to the theatre and they worked on me and they give me my baby.”

**Interviewer: Q2 Tell me about your experiences when you were referred?**

**Respondent 9:** “my experience was good because I was sure I will have my baby if I reach ........ as soon as they gave us the letter, we came with a boda-boda here but I was worried on the way because the humps might make the baby to come very fast, we had to move slowly since the pain was much and also the water was coming so we took long on the road and we arrived at around 2pm. Thanks be to God we arrived well.”

**Interviewer: Q3 When you were told, you are coming to ......., did you have any reservations/reluctance on referral? What were your thoughts about referral?**

**Respondent 9:** “I did not have any fears because I said whenever I cry or whenever I do what, I must deliver my baby, then they transferred me this way, now not even making any shaking or making that thinking bad things because it will make things worse, so I have to just accept the situation so long as I have my baby.”

**Interviewer: Q4 What do you think about the care of mothers from where you were referred? Would you rate the quality of care where you were referred?**

**Respondent 9:** “At .......? (pause) they are just, they don’t care well. The sick might come, they just say today am not on duty, the in-charge told them you must work. This one says am not on duty today, they ask another and it is the same, even patients are not there because the staff are never there always, they are just there, there. Even if you want medicine, they want money and the government gives them, they want money, not to go a buy medicine. If you have money, they work on you very fast, if you don’t have money, they just look at you and leave you alone.” If am to rate the quality of care, I would just say it is fair.

**Interviewer: Q5 What do you think about the quality of care you have received from here?**

**Respondent 9:** “The care is good. The care given to mothers is very good, whenever you want this, they work on you, whenever you don’t have money, they might pick the medicine from the pharmacy then write for you the rest to buy when you get the money but at ....... that is not the case. They also keep on monitoring and checking pressure, asking you how you feel, and give you the pain killers.”

**Interviewer: Q6 Was this the place you were referred to?**

**Respondent 9:** “Yes, when they told me that am going to be transferred, they said ‘we transfer you to .......HC-IV or ....... Hospital?’ I told them I want to go to ....... because my father works there and at ....... they care for mothers very well and even other patients, they work well. So, they wrote the letter and they gave me.

**Interviewer: Q7 What did you like about referral?**

**Respondent 9:** “I like referral because they make you to choose where to go and also because my baby refused to come from there but when I was referred, I had my baby through operation, and another thing is because here the staff work well on mothers that is why I like this way and referral.

**Interviewer: Q8 What did you not like about being referred?**

**Respondent 9:** “What I did not like? (pause) referral was good but here! There is some smell outside there were we sit, the cleaning is not okay outside the ward, people urinate out at night, there is also some blood near the rubbish bucket. The cleaners are very tough when they are cleaning the ward”.

**Interviewer: Q9 What has been your overall experience when you were referred?**

**Respondent 9:** “my experience I can say was good because being a first-time mother is that, labor is very painful but I just say to myself I must deliver my baby even if they operate me. I wish if they had transferred me the very day, I went to ......., I would have not suffered this pain.

**Interviewer: Q10 What was your experience on the road during referral?**

**Respondent 9:** “On the way? I came by Boda-boda (motorbike). What I liked is that the Boda-boda when they are carrying you (pregnant mother) they make sure that they reduce the speed when they reach the humps and also that helps the baby to go down inside. The road is good because it is tarmac and not congested, so we reached safely.

**Interviewer: Q11 What was your experience when you were received here? What was the quality of care you received here?**

**Respondent 9:** “here the received me well, asked me for a kavera (polythene sheet) to put on the bed and they checked on me immediately and they said am going to deliver. but it reached Saturday and they said ‘am going to be operated’ and I willingly accepted because I must deliver my baby. Then they wrote for us to buy some things for the theatre like jik, Omo and some medicine

**Interviewer: Q12 how were you handled when you arrived here in ....... Regional Referral Hospital?**

**Respondent 9:** “I was handled well. I came here they went I checked me well, still they said that am 6 cm then they told me to come back at 7 pm for checking. I went and still it was 7 cm then the doctor told me to come at 8 pm, I went there and they said that it was 5 cm, then I started worried. I went out and I said to myself what is this? The baby is till up but am feeling much pain. whenever I get worried it cannot change anything I must deliver my baby. They then put for me a catheter for urine and they told me let us wait tomorrow. My father said let me just talk to the doctor and they transfer you to the theatre. Then my father said ‘are you willing to go to the theatre?’ I told him that am willing because I need to remove the baby (Pause). I will make the baby tired. They then put me some water (drip). Aaya, I waited and waited, it reached 10 am and they said ‘many people are still there’, it reached 2 pm and they took me to the theatre, they worked on me and they removed the baby when it was alive, now I have my baby.”

**INTERVIEW DETAILS:**

**Interview time:** 15 minutes:05 seconds

**Interview location:** Postnatal ward (.......)

**Moderation language:** English

**Participant number:** 10

**Conversation language definitions:**

**Bold:** Title/interview questions

**Normal text:** Respondent

**Silent talk:** (Pause)

**I:** Interviewer

**R:** Respondent/participant

**Q:** Interview question

| Demographic data: | Response |
| --- | --- |
| Initials  Age:  Marital status: Married  Education level  Parity  Source of referral  Contacted the receiving facility prior to referral  Mode of transport  Distance from the referring facility  Mode of delivery  Outcome of baby after delivery | WN  23 years  Married  S.4  1  ....... HC-III -....... district  No  Boda-Boda  49.2 km  Caesarean section  Alive with the mother |

**RECORDED INTERVIEW**

**Interviewer: Q1 Tell me about your delivery?**

**Respondent 10:** “actually I was here like for three days eeh, I was like I fell sick with malaria. I finished like three days then from there I went back home to ........ Reaching there that very day is when labor started at around 10 pm until the next day in the morning then I went to the health centre III on Wednesday (01/11/2023) reaching there I found no doctor or nurse. I don’t know actually the place is remote but everything is there only that I found there is no health worker. I stayed there like for three hours then a certain nurse came (Midwife) she checked me and told me it is okay, do exercise, I said it is okay. I moved, I moved, it reached an extent she called me again she checked me and told me actually I should give you a referral because I am the only health worker who is here, no one is there to help me so I should just refer you to ....... HC-III. I called my husband, my husband works in town here then my husband told me, no don’t stop at ....... just come to ....... regional hospital then I came.

I left there at around 12 noon and reached here at around 2 pm because it had rained you know a remote place. Reaching here I found health workers actually they helped me they try their level best they helped me they checked me and told me your time is still, I was at 8 cm then they told me I will be checked at 8 pm again. The time reached and still I was at 8 cm. they told me you have to go up to 10 pm. I did an exercise, I did exercise. Reaching 10pm they told me aah you still have time you relax like an hour. I relaxed, reaching 11pm there, the doctor came and checked me and said the baby is fat so what to do, you just go an operation.”

**Interviewer: Q2 Tell me about your experiences when you were referred?**

**Respondent 10:** “of course I was actually affected because it had rained, the weather was not good because I was using a motorbike, but we tried not until we reached the junction to Kapchorwa where tarmac is now there, I felt a lot of pain.”

**Interviewer: Q3 When you were told, you are coming to ......., did you have any reservations/reluctance on referral? What were your thoughts about referral?**

**Respondent 10:** “I didn’t feel bad about referral because I just new it was a health centre three and they are referring me to a health centre -IV. I know that everything is going to be okay.”

**Interviewer: Q4 What do you think about the care of mothers from where you were referred? Would you rate the quality of care where you were referred?**

**Respondent 10:** “when I went to ....... health centre -III, I found patients and mothers there, no health worker they are just meandering around waiting for the health workers. They open the health centre very early for patient who come just waits. Actually, the time we were referred, I found one mother on the way, that they told her there is no medicine, just written for her to go and buy. That very one midwife in the maternity room was the one working again on the other patients.”

**Interviewer: Q5 What do you think about the quality of care you have received from here?**

**Respondent 10:** “actually if I compare everything was excellent because last time as I had told you I fell sick with malaria, they brought me up to here when I was pregnant. I was at ....... there they treated me from a certain clinic there then my mum called me, I went to ....... reaching there, she told me you will go to Kapchorwa main hospital. We went there and the situation was not good actually there. My husband then called me and said aah, aah you just come to ........ Reaching here actually the way they handled me here; I finished three days and I was fine. I even went back home. Then on reaching, home that is when the labor started then I came back again it means actually the care here is excellent.”

**Interviewer: Q6 Was this the place you were referred to?**

**Respondent 10:** “actually I was referred to ....... Health Centre -IV, then my husband said I should not stop there at ....... I should come straight to ....... Referral hospital.”

**Interviewer: Q7 What did you like about referral?**

**Respondent 10:** “what I have liked about referral? Of course, let me say that at the health facility where they have referred you to, may be that facility the services might be better than this one hmm, that is what I have liked about referral.”

**Interviewer: Q8 What did you not like about being referred?**

**Respondent 10:** “what I have not liked? Actually, nothing I have seen bad here hmm. I can say everything is okay. But from ....... health centre -III, it was only one midwife attending to everyone in the facility and that I did not like.”

**Interviewer: Q9 What has been your overall experience when you were referred?**

**Respondent 10: “**it was actually good and excellent just because I had tried Kapchorwa Hospital I told you when I had malaria before coming here, the way they handled me here is much better to what I experienced in Kapchorwa Hospital. That is why I say here is better compared to the other ne.”

**Interviewer: Q10 What was your experience on the road during referral?**

**Respondent 10:** “I was feeling very bad on the road, because I used a motorbike (Boda-boda). first it had rained and the road was very bad from ....... it is Marram and the trucks are working on the road until we joined the tarmac at the junction from the road going to Kapchorwa. Also, now the humps actually made the pain even more along the road.”

**Interviewer: Q11 What was your experience when you were received here? What was the quality of care you received here?**

**Respondent 10:** “actually if I compare everything here is excellent because last time as I had told you I fell sick with malaria, they brought me up to here when I was pregnant. So, I still had the comfort and I had to come back here for delivery because I know they will care for me very well. And they indeed cared and am happy.”

**Interviewer: Q12 how were you handled when you arrived here in ....... Regional Referral Hospital?**

**Respondent 10:** “when I arrived here, they handled me in a good way, actually they started working on me. I did not even spend 5 minutes before the staff came to work on me. After checking, they told me ‘You are going to deliver but just time was still’.”

**INTERVIEW DETAILS:**

**Interview time:** 20 minutes:20 seconds

**Interview location:** Postnatal ward (.......)

**Moderation language:** English

**Participant number:** 11

**Conversation language definitions:**

**Bold:** Title/interview questions

**Normal text:** Respondent

**Silent talk:** (Pause)

**I:** Interviewer

**R:** Respondent/participant

**Q:** Interview question

| Demographic data: | Response |
| --- | --- |
| Initials  Age:  Marital status: Married  Education level  Parity  Source of referral  Contacted the receiving facility prior to referral  Mode of transport  Distance from the referring facility  Mode of delivery  Outcome of baby after delivery | KJM  20 years  Married  S.4 (hair dressing)  1  ....... HC-IV  Yes  Taxi  28.3 Km  Caesarean section  Alive in Neonatal unit |

**RECORDED INTERVIEW**

**Interviewer: Q1 Tell me about your delivery?**

**Respondent 11:** “At first, I have been pouring water for almost one week and I went to hospital, they gave me some treatment. They first checked me that they wrote in my book first go and check for UTI and Malaria. They go UTI was not there. They go only malaria and it was not too high. They gave me the tablets Panadol and co-artem to swallow, after swallowing, they gave me Ampicillin injection that to stop the flow of water and they gave me 4 of them morning, 4 lunch and 4 evening. After three days, the other original nurse who first attended to me came and said, eeh! This girl made a mistake, she was supposed to give you two-two oba after 6 hours or? And she said if she comes again, tell her to give you only two. After that dose and treatment, they wrote the tablets there ampicillin again for me to swallow then I went home after discharge. But water was still going. I did not see any change even, as if they were just adding. After one week again aah mama told me oba! We go for scanning? I said if you go for scanning, they will shout at you there without a doctor’s signature eeh, that why did you come here for scanning when they have not indicated here, so I refused and money was not even there for the scan.

Now the third week, I poured, I poured. At night, the stomach started paining me. On that Friday morning, my condition was not good. I called mama, aah! Is like the stomach is paining me and I am pouring water! Smelly one and I was still at home. I packed my things and I went back to hospital back to ....... HC-III and they started checking-checking me there and there started paining because they were over checking me and they were like, this one is bragging, we have to refer her to ....... and yet they were not even giving me the correct result, they were like your baby is fine yet I was pouring water, that the baby is breathing well and I was pouring smelly water eeh so after there at night the other nurse came checked me again, there was over paining. It was paining me totally because of over checking. Me I was like nurse give me some few minutes and the other nurse started abusing me that I am going to refer you to ......., let me go and sleep.

At around midnight, I was feeling stomach pain even getting the head up was a problem and the baby was coming up like this, not the usual playing, aah I was feeling too much pain! And they told me to over walk, that if you don’t over walk, you will not give birth, you over walk, keep on walking and take tea. I took ten (10) cups of tea and nothing was even happening.

Reaching in the morning, the nurse was like if you don’t want to be checked, I am going to refer you. They took me there again for checking at around 7 am she pushed her hand there and the moment she pushed her hand, she saw blood, even me I saw with my eyes blood coming out plus smelly-smelly water! As if that water was mixed with flue (Mucus). She was like my hand had nt even reached there and you are panicking. Me I was like aah-aah it is now enough for me it is paining and I am feeling too much pain and she left me there that you collect your paper and go way from there. She went and said let me write the referral form now for you to leave this place. She wrote and for me I went to ....... HC-IV.

Before we left, she called the other nurse at ....... telling her on phone that she has referred two girls there but these girls they don’t want to be checked and they have bad manners. We used a boda-boda and I was feeling a lot of pain on the way. So, when we reached there, the nurse was like they told me your problem, that you don’t want to be check. I explained to her. She was like okay sleep on the bed and I check you. The nurse checked me very well and I did not even feel the pain. She placed the machine on my stomach and the machine started crying then she said, this child was left with only 30 minutes so there is no way I can help you here you have to go to Ongino Hospital oba .......?

But my sister said aah at least you write ........ So, she wrote ........ Then we got a taxi because they said that the ambulance cannot run to ........ It runs in this side of Kumi only. I did not even see the ambulance around so we got a taxi up to here and I was feeling too much pain on the way even the back, the waist, down there and I was pouring smelly water. They did not even escort me. I just came with my sister and mama.”

**Interviewer: Q2 Tell me about your experiences when you were referred?**

**Respondent 11:** “anti-they checked me. At first from ......., they were telling me here is small I cannot push the baby. I came here and they told me we are going to operate you, your baby is even tired they delayed with you and at that time, I was feeling too much pain.”

**Interviewer: Q3 When you were told, you are coming to ......., did you have any reservations/reluctance on referral? What were your thoughts about referral?**

**Respondent 11:** “oh no I did not have any fears of coming here, me, I was stable because I had suffered for long with pouring water and stomach and I wanted to be fine and to rest although I had too much pain and in fact, they would have done this earlier.”

**Interviewer: Q4 What do you think about the care of mothers from where you were referred? Would you rate the quality of care where you were referred?**

**Respondent 11:** “the care there most of the time you have to just force them to treat you, when the time reaches for treatment, you can’t see any nurse, you just follow them to their homes to come and treat you and they will start telling you bad words even before you leave their homes. The response is like aah, we are tired you people ae also disturbing us aah am going to check this one only and come back.”

I can say the care there is poor because the way they handled me was not good. It is poor even when they were giving me treatment to stop the water we went there with mama and they told us to buy everything including even the plaster, the cannulas, medicine at .......! And when the time reaches, you just call them and say nurse now it is time for treatment and sometimes the other one can just jeer at you before coming, so that thing hurts me a lot.”

**Interviewer: Q5 What do you think about the quality of care you have received from here?**

**Respondent 11:** “the care here is totally good. The moment I reached here, when it reaches times for treatment, the nurses and doctors are caring for their patients, they are over asking that how are you feeling, but the other side, no one can even ask you. You just have to follow them up. The interaction between the mothers and the health workers here is good. Even in the morning they come and greet, and ask ‘how are you feeling now’ and if you explain to them, they give you immediate care.”

**Interviewer: Q6 Was this the place you were referred to?**

**Respondent 11:** “no. first from ......., they referred me to ....... then from there they referred me to Ongino Hospital until my sister requested the nurse to write a referral to at least ....... and that is how we came here.”

**Interviewer: Q7 What did you like about referral?**

**Respondent 11:** “I have like referral because it saved my life because the three weeks, I had been pouring water I could not imagine I would have my baby with me, now I have got her

**Interviewer: Q8 What did you not like about being referred?**

**Respondent 11:** “I did not like referral because of the many referrals from here to there they could not make one decision of one hospital where I can get the service at once and also, I did not like the way some nurses can scare mothers putting a bad picture ahead of the place they are referring you to, may be to discourage you or to cover their arrogance and also the comments made ‘like, this one is bragging, we have to refer her to .......’.”

**Interviewer: Q9 What has been your overall experience when you were referred?**

**Respondent 11: “**my experience about referral was not good because the midwife there was not willing to refer me, I felt I was a burden to her and she referred me because I was not happy and I was complaining and telling her about how the examination was painful.”

My experience was not good in the beginning but later it became good because they referred me to get help very fast because the pain was too much, even me I am praying if there was a way these people can help me instead of dying here in ........ They were like even if you go there, they are going to check you the very way. There is even worse, they tie you with ropes, they slap, they beat you and me I was not even scared of what she was saying, I was there just keeping quite.”

**Interviewer: Q10 What was your experience on the road during referral?**

**Respondent 11:** “aah-aah the pain was too much and I was feeling too hot in the taxi, the all body was hot and the pain was too much. I have been just laying behind there they gave me all the chairs behind the taxi to lay.

**Interviewer: Q11 What was your experience when you were received here? What was the quality of care you received here?**

**Respondent 11:** “I cannot remember the time we arrived here oba around 11 am, for sure that time my brain was not working because I was in too much pain. As I had told you the care here is good and the nurses and doctors are concern about their patients.” it took oba 3 hours for me to go to theatre because they gave me some medicine first in drip the moment, I reached they gave me serious attention, they put me that thing for urine but the stomach pain did not stop.”

**Interviewer: Q12 how were you handled when you arrived here in ....... Regional Referral Hospital?**

**Respondent 11:** “They handled me carefully they checked me immediately on arrival they put me on I drip of fluid with some medicine and they told me to wait until it is finished. To me I was in much pain and was thinking like if there was a way it could get finished fast and the doctor comes and takes me to the theatre for operation so that I can rest very fast.”

**INTERVIEW DETAILS:**

**Interview time:** 13 minutes:45 seconds

**Interview location:** Postnatal ward (.......)

**Moderation language:** English

**Participant number:** 12

**Conversation language definitions:**

**Bold:** Title/interview questions

**Normal text:** Respondent

**Silent talk:**(Pause)

**I:** Interviewer

**R:** Respondent/participant

**Q:** Interview question

| Demographic data: | Response |
| --- | --- |
| Initials  Age:  Marital status: Married  Education level  Parity  Source of referral  Contacted the receiving facility prior to referral  Mode of transport  Distance from the referring facility  Mode of delivery  Outcome of baby after delivery | KH  29 years  Married  S.3  4  ....... HC-III  No  Boda-boda  5.5 km  Caesarean section  Alive in neonatal unit |

**RECORDED INTERVIEW**

**Interviewer: Q1 Tell me about your delivery?**

**Respondent 12:** “My delivery was not easy because I came, it was on Wednesday I was in labor but I sat here until Friday evening. The doctor told me you first wait we treat the pressure and see. If it comes down, we take you to theatre, so I waited until Friday evening I entered theatre at around 4 pm and came out at around 6 pm. So, my delivery was not easy.

**Interviewer: Q2 Tell me about your experiences when you were referred?**

**Respondent 12:** “Hmm, hmm, I was not feeling well because it was my first time to come this way, I have been delivering my children from ....... HC-III. So, when they told me about referral, I was even scared, I started crying because I said if I reach there, the next thing is just operation. I was scared of the operation. I am even wondering I went there to the theatre and came back alive? God does wonders.”

**Interviewer: Q3 When you were told, you are coming to ......., did you have any reservations/reluctance on referral? What were your thoughts about referral?**

**Respondent 12:** “No I came immediately because the nurse told me if you go home you don’t sleep, just make sure you go to the hospital because the baby is already near but it is just the pressure which is high. You stay at home; you may lose the baby.”

**Interviewer: Q4 What do you think about the care of mothers from where you were referred? Would you rate the quality of care where you were referred?**

**Respondent 12:** “the care of mothers there is not bad, even the health workers are not bad, because they do not talk badly, they are friendly with the mothers as they care, they ask you, and they treat you very well.”

**Interviewer: Q5 What do you think about the quality of care you have received from here?**

**Respondent 12:** “here? they treated me very well because they are not tough, they talk to you very well as a sick person, they are friendly, they ask you how you are feeling and when you ask them something, they bring for you, if you tell them, I am feeling pain, or this and that, they care and treat you. So, they are not hard, they are friendly.”

**Interviewer: Q6 Was this the place you were referred to?**

**Respondent 12:** “yes, this was the place I was referred to as the nurses advised me, so I did not change or think of another place again.”

**Interviewer: Q7 What did you like about referral?**

**Respondent 12:** “Eeh aah, at first, I feared so I was scared. I hear people there complaining about the nurses of ....... main hospital that are hard, they do what they want. But me I have seen them; they are not what I hear of and expected of them! They are not like the way I heard, they are very good and even the place is good.”

**Interviewer: Q8 What did you not like about being referred?**

**Respondent 12:** “aah, what I can say is like these beds are too high for people to climb because when you are operated, even the legs are heavy and it is difficult to lift/raise since the pain is much. At least they should bring for us these short ones.”

**Interviewer: Q9 What has been your overall experience when you were referred?**

**Respondent 12:** “Hmm the care every time. They come and check on you, they ask you how you are feeling and how you are coping with the pain.”

**Interviewer: Q10 What was your experience on the road during referral?**

**Respondent 12:** “Actually me I came by boda-boda because I came from home after the nurse told me to make sure that I come to hospital. Th road was bad because from my home to main road is not having tarmac and it had rained so the road was bad and also the humps were there and holes on the road.”

**Interviewer: Q11 What was your experience when you were received here? What was the quality of care you received here?**

**Respondent 12:** “They welcomed me well because the main problem was high pressure. The doctor told me you first wait we treat the pressure and see. If it comes down, we take you to theatre, but it failed to come down so they had to take me for operation after two days of waiting.”

**Interviewer: Q12 how were you handled when you arrived here in ....... Regional Referral Hospital?**

**Respondent 12:** “they handled me very well because I came late, but I met one doctor and I asked him and he asked me ‘where is your antenatal card, book and the referral form? I had and I gave him. After giving him, he took where the doctor was and that doctor called me that very time. She checked on me and told me you first wait and we first treat the pressure. After two days if the pressure refuses, we take you, if not you can push. But still the pressure refused to come down so she decided to take me to theatre because now the baby was at risk.”

**INTERVIEW DETAILS:**

**Interview time:** 18 minutes:14 seconds

**Interview location:** Postnatal ward (.......)

**Moderation language:** English

**Participant number:** 13

**Conversation language definitions:**

**Bold:** Title/interview questions

**Normal text:** Respondent

**Silent talk:** (Pause)

**I:** Interviewer

**R:** Respondent/participant

**Q:** Interview question

| Demographic data: | Response |
| --- | --- |
| Initials  Age:  Marital status: Married  Education level  Parity  Source of referral  Contacted the receiving facility prior to referral  Mode of transport  Distance from the referring facility  Mode of delivery  Outcome of baby after delivery | NZ  23 years  Cohabiting  Tertiary institution  1  ....... HC-IV  Yes  ambulance  3.7 km  Caesarean section  Alive in neonatal unit |

**RECORDED INTERVIEW**

**Interviewer: Q1 Tell me about your delivery?**

**Respondent 13:** “For my first time, I attended my antenatal care at ....... Health Centre-IV where I got my antenatal care. I stared when the pregnancy was three months up to eight months. Therefore, at seven months I went to ....... Health Centre-IV and they sent me for a scan to ....... Regional Referral Hospital because they do not do scanning there and I even have the results of the scan. They told me on the scan that I will produce on 30^th^ /09/2023, therefore it past the days of the scan up to 12^th^/10/2023 is when I started my labor pain and I went to ....... Health Centre- IV for delivery and they told me that I was going to push and the way was so clear.

I started feeling signs of delivery at around 5 pm and they told me that the baby was at 5 cm when they checked me and they told me that I am going to deliver well so it is only remaining hours and I started to push the baby at around 8 pm and the baby’s head was just showing and going back, showing and going back! I did not know the reason why it was doing that. They tried more to make me deliver because they were seeing the way widening up to make me not to be operated so that I can deliver normally and they tried from around 8 pm – 4 am and I failed. Failing, the doctor told me that let my call my friends in ....... so that they can help you to push the baby, you are capable of pushing but the I don’t know why? Because they may do better than me because me, I have failed. But the doctor told me that I have the energy but the pressure (force) is not enough to push and she told me that the baby is big. She said let me try to make sure that the pressure is increased. Then she said you go to ....... Regional Referral Hospital. Let me call up my friends so that they can come and pick up and they sent an ambulance to come and pick me at around 4:20 am and I came to ....... main hospital.

Reaching ....... Regional Referral Hospital, they first checked on me and said that the baby is in a position of coming out and I am capable of pushing it and they told me to relax on the side way so that the baby is safe and I increase on the pressure on my pressure of pushing. Therefore, they first waited up to 11 pm I was pushing but the was failing to come out. they told me let us help you, you have enough strength to push but the baby is too big. Then I told them you do operation to me because the pressure is the one making me not to push. They said, you try – you try several times they wanted me just to push but I failed. They measured each and everything it was fine but I just surrendered my life and I said let me do operation. They again said you have to push – you have to push.

After telling me like that, they were just rotating around me saying that you are going to push and I told them let me do operation and they accepted and said aah, if you have accepted to do operation, let us call the doctor for the operation and he comes and works on you. Then I signed up the forms for the operation and they did to me operation during day time.”

**Interviewer: Q2 Tell me about your experiences when you were referred?**

**Respondent 13:** “my referral was good because even from ....... and here the doctors wanted me to push. Even the way was enough for the baby to pass but the pressure was not enough and the baby was also big being my first baby, I had hope that I will deliver when I reach here because the head was just near but the pressure failed me.”

**Interviewer: Q3 When you were told, you are coming to ......., did you have any reservations/reluctance on referral? What were your thoughts about referral?**

**Respondent 13:** “when I was told about referral, I did not have any fears of coming because I was in the time of delivering and I had hope that I am going to deliver because the doctor there tried to make me deliver but the pressure made me fail since it was not enough to push the big baby.”

**Interviewer: Q4 What do you think about the care of mothers from where you were referred? Would you rate the quality of care where you were referred?**

**Respondent 13:** “the care there is quite good, they can help on the mother during antenatal care plus they can give much care about your complaints, about what you are experiencing. If it needs anything, they can give you like mosquito nets, mama kits. They try their best but ....... Main Hospital is better than ....... HC-IV.”

**Interviewer: Q5 What do you think about the quality of care you have received from here?**

**Respondent 13:** “I have received much care from here. They just operated me for free, they were giving me medicine plus some other medicines I was buying from outside but they gave me what they had. I was buying the medicine when it was not in stock but when it is there they give.”

**Interviewer: Q6 Was this the place you were referred to?**

**Respondent 13:** “yes I was referred here to ....... main hospital because the doctor there called her friends here to help me and actually when I arrived the helped me.”

**Interviewer: Q7 What did you like about referral?**

**Respondent 13:** “The way they have worked on me, it was good, they showed me much love and care but I just failed to push the baby. But they were telling me I was capable of pushing the baby. I have also liked the way the nurses and doctors were giving me time to push, they did not rush me to operation because they said I was capable of pushing so I had hopes of delivering but it failed.”

**Interviewer: Q8 What did you not like about being referred?**

**Respondent 13:** “Hmm-hmm, nothing because they gave me everything. Everything was fine even the staff.”

**Interviewer: Q9 What has been your overall experience when you were referred?**

**Respondent 13: “**....... Main Hospital is better than ....... HC-IV. I have got experience that if I get another baby, I will just come to ....... main Hospital because it has much care.

**Interviewer: Q10 What was your experience on the road during referral?**

**Respondent 13:** “After calling the ambulance, it took only about 3 – 5 minutes after we left ........ It did not take even a long time and also reaching here, they just worked on me immediately because they were informed and they had all the information about me that I was coming.”

**Interviewer: Q11 What was your experience when you were received here? What was the quality of care you received here?**

**Respondent 13:** “Here I was received very well and they worked on me immediately because the other doctor had informed them and they were ready to help me and they showed me much care when I arrived here and they gave me hope of delivering but the pressure let me down. They were kind and encouraging me and they kept around me to encourage and comfort me.”

**Interviewer: Q12 how were you handled when you arrived here in ....... Regional Referral Hospital?**

**Respondent 13:** “They welcomed me and they showed me much love and care, they were giving me hope for delivering, but I just failed to push the baby.”

**INTERVIEW DETAILS:**

**Interview time:** 13 minutes:32 seconds

**Interview location:** Postnatal ward (.......)

**Moderation language:** English

**Participant number:** 14

**Conversation language definitions:**

**Bold:** Title/interview questions

**Normal text:** Respondent

**Silent talk:** (Pause)

**I:** Interviewer

**R:** Respondent/participant

**Q:** Interview question

| Demographic data: | Response |
| --- | --- |
| Initials  Age:  Marital status: Married  Education level  Parity  Source of referral  Contacted the receiving facility prior to referral  Mode of transport  Distance from the referring facility  Mode of delivery  Outcome of baby after delivery | NJ  31 years  Married  S.4  4  ....... HC-IV  Yes  Boda-boda  3.7 km  Caesarean section  Alive with the mother |

**RECORDED INTERVIEW**

**Interviewer: Q1 Tell me about your delivery?**

**Respondent 14:** “My delivery wasn’t easy because my labor I first saw a lot of blood, I was bleeding a lot then I had to come to hospital. Reaching here they told me I was at 8 cm, then after some time again they said I was 6 cm. I found it difficult and, in the morning, they conclude that the baby is too big they are going to operate me and then they wrote me a referral form and I had to go home then came to ....... referral that very day. Normally I have been delivering big babies, my first born was 5.8 kgs, the 2^nd^ was just 3.5 kgs, 3^rd^ 4.8 kgs and now this one is 5.5 kgs. And this is the first time I have been operated.

**Interviewer: Q2 Tell me about your experiences when you were referred?**

**Respondent 14:** “yeah, I even first of all felt they are going to operate on me that was the first thing that came to my mind. I was Soo scared because they told me first go and check with the doctor. I said again what? May be my baby is not alive, but the doctor told me no, just the baby is too big we want you to be near, ....... now days they don’t do operations so it is better to go where in case of any failure, they can do operation.”

**Interviewer: Q3 When you were told, you are coming to ......., did you have any reservations/reluctance on referral? What were your thoughts about referral?**

**Respondent 14:** “I did not have reservations; I was willing to come because I had no other option but to have my baby alive in whatever way.”

**Interviewer: Q4 What do you think about the care of mothers from where you were referred? Would you rate the quality of care where you were referred?**

**Respondent 14:** “they care very well; their care was good for example my 3^rd^ born I delivered from there and I was so cared for. I was almost again failing but they tried their best until I just had a normal delivery. Their care was good for me personally. I don’t know exactly why they don’t do operations these days in ........ They used to do them but they said oba one of the machines have a problem I don’t know. So, they transfer all patients this way.”

**Interviewer: Q5 What do you think about the quality of care you have received from here?**

**Respondent 14:** “from here also the care wasn’t bad, it was also good because they managed to operate on me and now, I am alive with my baby and that was a very good care to me. The nurses and the doctors are also good only that the problem is the tribes (languages), some come when they know only lugisu, no it becomes a problem if you don’t know that language.”

**Interviewer: Q6 Was this the place you were referred to?**

**Respondent 14:** “yeah this was the place I was referred. And if there was an option to choose, I will chose ....... regional referral because they offer good services to mothers and also it is near my home.”

**Interviewer: Q7 What did you like about referral?**

**Respondent 14:** “I have liked referral because from here incase of any failure in delivery, there is an option faster-faster, the theatre is just next to the labor suite. I have also liked their treatment.”

**Interviewer: Q8 What did you not like about being referred?**

**Respondent 14:** “there was nothing I saw wrong in referral because at least they saved my life and that of the baby, and may be also the in-charge of ....... should find how to work on the machines there so that mothers can be worked there.”

**Interviewer: Q9 What has been your overall experience when you were referred?**

**Respondent 14: “**my experience about referral is that their treatment is good and the care a lot for the mothers generally is good.”

**Interviewer: Q10 What was your experience on the road during referral?**

**Respondent 14:** “Our road was too bad because I came by Boda-boda. Namabasa to this way is not tarmac. The holes are too much on the road and when it rains ha-ha, and also when you come to deliver you are in that pain and the road is also adding on it especially the holes and the humps.”

**Interviewer: Q11 What was your experience when you were received here? What was the quality of care you received here?**

**Respondent 14:** “The quality of care here is excellent because they worked on me being my first operation, I had fear and was not expecting to come back alive with my baby, but glory to God here I am alive.”

**Interviewer: Q12 how were you handled when you arrived here in ....... Regional Referral Hospital?**

**Respondent 14:** “I was handled quickly as soon as I reached. The nurses were there, they received me faster and worked on me. They encouraged me, they told me you keep around you are going to deliver.”

**INTERVIEW DETAILS:**

**Interview time:** 14 minutes:32 seconds

**Interview location:** Postnatal ward (.......)

**Moderation language:** English

**Participant number:** 15

**Conversation language definitions:**

**Bold:** Title/interview questions

**Normal text:** Respondent

**Silent talk:** (Pause)

**I:** Interviewer

**R:** Respondent/participant

**Q:** Interview question

| Demographic data: | Response |
| --- | --- |
| Initials  Age:  Marital status: Married  Education level  Parity  Source of referral  Contacted the receiving facility prior to referral  Mode of transport  Distance from the referring facility  Mode of delivery  Outcome of baby after delivery | NJ  30 years  Married  S.4 (Nursery Teacher)  2  ....... HC -IV  Yes  Taxi  24 km  Caesarean section  Alive with the mother |

**RECORDED INTERVIEW**

**Interviewer: Q1 Tell me about your delivery?**

**Respondent 15:** “I started my labor on Thursday at home, I stayed there feeling pain, pain, back pain and am at home. So, on Friday, I went to ....... HC-IV they checked me and they said you are a doctor’s case because you have a fresh previous scar and you cannot deliver here because of high risk. They wrote for me referral to come to ....... RRH. So, I had to go home to organize my self to come to ........ I came here on Sunday by Taxi at around 1pm. I reached here but they didn’t work on me at around that time, I found those who were working day time when they were planning to leave so I stayed there. It was at 6pm when they started to examine me, so after checking, they told me am supposed to be operated, the reason being because of a fresh scar. So, the whole night I was here waiting and connecting to the doctor until yesterday when they operated me.”

**Interviewer: Q2 Tell me about your experiences when you were referred?**

**Respondent 15:** “I felt I wasted my time going to ....... HC-IV because my case was already known to me, I would have just come here straight. I reached here although they delayed, I was handled well in the theatre, the doctors stay there and continue talking to you and giving you hope.”

**Interviewer: Q3 When you were told, you are coming to ......., did you have any reservations/reluctance on referral? What were your thoughts about referral?**

**Respondent 15:** “When they told me that am going to be operated, the first think that came to my mind was I felt that, I wish if I could reach there and deliver before the operation, that is the first one and it was my prayer. I was not willing to do the operation and I also had a fear to reach there that is why I first went home but I just said God have mercy on me, am not the one who wanted it.

**Interviewer: Q4 What do you think about the care of mothers from where you were referred? Would you rate the quality of care where you were referred?**

**Respondent:** “I think the care is good because even me they explained to me why I have to deliver in the hospital since I had a fresh scar, and really those nurses attend to mothers. Only that for me I was hoping if I was to try to deliver but they insisted I should go to the main hospital so now here I am because they cared.”

**Interviewer: Q5 What do you think about the quality of care you have received from here?**

**Respondent 15:** “The care here is not all that bad, because they are working since the time they operated me, they are checking on me they are treating me. Now I am from receiving other medicines. During morning, I was feeling too much pain, now am from receiving treatment and it is somehow reducing and there is a change. So, I can rate the care to be good.”

**Interviewer: Q6 Was this the place you were referred to?**

**Respondent 15:** “yes anti-when I went to ......., they checked my ANC and said this is a doctor’s case and it is for hospital delivery and they cannot do anything so then they asked me and wrote for me a referral letter to come with to ....... due to fresh scar.”

**Interviewer: Q7 What did you like about referral?**

**Respondent 15:** “The referral? I have like the referral because, from the other side when you stay, it is very far and when time reaches and you have a problem, it becomes difficult to care for you in terms of transport to bring you here, so referral is good such that one can be near the main hospital. I have also like referral because they tell you the right thing and then before so that your life and the baby are not at risk.”

**Interviewer: Q8 What did you not like about being referred?**

**Respondent 15:** “I have not liked referral era! again because of transport money. I come from very far, I put in transport of 10,000/= to and from so have not liked that because at ......., I just move to the health centre at no cost.”

**Interviewer: Q9 What has been your overall experience when you were referred?**

**Respondent 15: “**my overall experience has been good because am alive and my baby is also alive. On referral at first, I was not willing to come, I first went home then I said God have mercy on me, it is not me who wanted. So, I put everything to God then I decided to come here with my husband.”

**Interviewer: Q10 What was your experience on the road during referral?**

**Respondent 15:** “As I had told you at first, I was not willing to come, so later when I accepted, I used a public transport (Taxi) there I had to wait for it to fill then they over squeeze people and also, they take long stopping over on the way. If I had accepted earlier, I would have used the ambulance instead. The road is good after joining the main road, but from ....... it is not good.”

**Interviewer: Q11 What was your experience when you were received here? What was the quality of care you received here?**

**Respondent 15:** “for sure it was not good because they took long to attend to me since there was handing over to the evening nurses and also, they first sent me to the laboratory to test then back to the outside laboratories yet I was feeling difficulty in moving. But the care later was good because the operation went well and they keep monitoring you while on the ward, when you report any pain, they attend to you immediately.”

**Interviewer: Q12 how were you handled when you arrived here in ....... Regional Referral Hospital?**

**Respondent 15:** “By that time I felt by the time I reach here they start to work on me immediately. By the time I reached, I found those ones who were working day when they have gone to lunch, when they came back, they were preparing to leave so they handed me to those ones for evening. After handing, I thought they were going to start that very day, so for them they started by sending me to the laboratory to go and test blood and urine and those ones in the laboratory were not working that time, they then told me to go out, so I was feeling difficulty to move from here to go and do the tests. After coming back, they told me that I have UTI in pregnancy and again they started treating me. Me I thought after testing they are going to operate me immediately but they started treating me first.”
